# Supplementary material for: Lipid metabolism of hepatocyte-like cells supports intestinal tumor growth in Drosophila
Source: Nat Commun. 2026 Jul 3;17:8296. doi: 10.1038/s41467-026-75074-w (PMC13470496; doi:10.1038/s41467-026-75074-w)
Supplement: Supplementary file 1 [file 41467_2026_75074_MOESM10_ESM.pdf]

nature portfolio

Corresponding author(s): Norbert Perrimon
Kerui Huang

Last updated by author(s): 04/09/2026

# Reporting Summary

Nature Portfolio wishes to improve the reproducibility of the work that we publish. This form provides structure for consistency and transparency in reporting. For further information on Nature Portfolio policies, see our Editorial Policies and the Editorial Policy Checklist.

## Statistics

For all statistical analyses, confirm that the following items are present in the figure legend, table legend, main text, or Methods section.

| n/a | Confirmed | |
|---|---|---|
| ☐ | ☒ | The exact sample size (*n*) for each experimental group/condition, given as a discrete number and unit of measurement |
| ☐ | ☒ | A statement on whether measurements were taken from distinct samples or whether the same sample was measured repeatedly |
| ☐ | ☒ | The statistical test(s) used AND whether they are one- or two-sided<br>*Only common tests should be described solely by name; describe more complex techniques in the Methods section.* |
| ☒ | ☐ | A description of all covariates tested |
| ☐ | ☒ | A description of any assumptions or corrections, such as tests of normality and adjustment for multiple comparisons |
| ☐ | ☒ | A full description of the statistical parameters including central tendency (e.g. means) or other basic estimates (e.g. regression coefficient) AND variation (e.g. standard deviation) or associated estimates of uncertainty (e.g. confidence intervals) |
| ☐ | ☒ | For null hypothesis testing, the test statistic (e.g. *F*, *t*, *r*) with confidence intervals, effect sizes, degrees of freedom and *P* value noted<br>*Give P values as exact values whenever suitable.* |
| ☒ | ☐ | For Bayesian analysis, information on the choice of priors and Markov chain Monte Carlo settings |
| ☒ | ☐ | For hierarchical and complex designs, identification of the appropriate level for tests and full reporting of outcomes |
| ☒ | ☐ | Estimates of effect sizes (e.g. Cohen's *d*, Pearson's *r*), indicating how they were calculated |

*Our web collection on statistics for biologists contains articles on many of the points above.*

## Software and code

Policy information about availability of computer code

| | |
|---|---|
| Data collection | CFX Maestro Software for Bio-Rad CFX Real-Time PCR Systems, Zeiss Zen Blue acquisition software, Olympus acquisition software. |
| Data analysis | See code for data analysis here: https://github.com/carriehuang1993/snRNAseq-analysis.git<br>Statistical testing was performed using Prism Version 10.2.0 (335), AutoTube (v 1.0), ImageJ 1.54f were used. |

For manuscripts utilizing custom algorithms or software that are central to the research but not yet described in published literature, software must be made available to editors and reviewers. We strongly encourage code deposition in a community repository (e.g. GitHub). See the Nature Portfolio guidelines for submitting code & software for further information.

## Data

Policy information about availability of data

All manuscripts must include a data availability statement. This statement should provide the following information, where applicable:

- Accession codes, unique identifiers, or web links for publicly available datasets
- A description of any restrictions on data availability
- For clinical datasets or third party data, please ensure that the statement adheres to our policy

Data generated and used in this study are described within the Article, Supplementary Data files, Supplementary Information, and Source Data file. Source data is provided in this paper.

## Research involving human participants, their data, or biological material

Policy information about studies with human participants or human data. See also policy information about sex, gender (identity/presentation), and sexual orientation and race, ethnicity and racism.

| | |
|---|---|
| Reporting on sex and gender | N/A |
| Reporting on race, ethnicity, or other socially relevant groupings | N/A |
| Population characteristics | N/A |
| Recruitment | N/A |
| Ethics oversight | N/A |

Note that full information on the approval of the study protocol must also be provided in the manuscript.

# Field-specific reporting

Please select the one below that is the best fit for your research. If you are not sure, read the appropriate sections before making your selection.

[x] Life sciences [ ] Behavioural & social sciences [ ] Ecological, evolutionary & environmental sciences

For a reference copy of the document with all sections, see nature.com/documents/nr-reporting-summary-flat.pdf

# Life sciences study design

All studies must disclose on these points even when the disclosure is negative.

| | |
|---|---|
| Sample size | Sample size was stated as the number of fruit flies used in each experiment. Sample sizes were determined based on previously published studies. For lipidomic analysis, sample sizes were determined based on this study (PMID: 38795635). For immunostaining of oenocytes, sample size was determined based on previously studies (PMID: 32523050, PMID: 33107824). For fly tumor related cachexia phenotypes, the sample size was determined according to the following studies PMID: 30639055, PMID: 25850671, PMID: 25850672. For trachea analysis, the following studies were used to determine the sample size: PMID: 33972730, PMID: 33972729, PMID: 38811741. For mice liver qPCR experiments, sample size was determined according to PMID: 29311302. Samples from the known group with the correct genotypes were selected randomly prior to experiments. |
| Data exclusions | No data was excluded from the analysis. |
| Replication | Each experiments were performed at least twice to verify the reproducibility. Some results are pooled from all replications. All attempts at replication were successful. |
| Randomization | N/A. |
| Blinding | No blinding was done. The investigators set up the experiments, collected and analyzed the data making it impossible to perform blind analysis. |

# Reporting for specific materials, systems and methods

We require information from authors about some types of materials, experimental systems and methods used in many studies. Here, indicate whether each material, system or method listed is relevant to your study. If you are not sure if a list item applies to your research, read the appropriate section before selecting a response.

## Materials & experimental systems

| n/a | Involved in the study | |
|---|---|---|
| [ ] | [x] | Antibodies |
| [ ] | [x] | Eukaryotic cell lines |
| [x] | [ ] | Palaeontology and archaeology |
| [ ] | [x] | Animals and other organisms |
| [x] | [ ] | Clinical data |
| [x] | [ ] | Dual use research of concern |
| [x] | [ ] | Plants |

## Methods

| n/a | Involved in the study | |
|---|---|---|
| [x] | [ ] | ChIP-seq |
| [x] | [ ] | Flow cytometry |
| [x] | [ ] | MRI-based neuroimaging |

## Antibodies

Antibodies used

1. Nb127D01-ALFA, 0.2mg/ml was used at a dilution of 1:500, made in this lab.
2. Rabbit-anti-pH3 (1:500, Cell Signaling, 9701)
3. Rabbit-anti-p4E-BP (1:800, Cell Signaling, 2855S)
4. Guinea pig-anti-dHnf4 (1:100, a gift from Dr. Gilles Storelli)
5. Rabbit Monoclonal Antibody HNF-4 alpha (1:1000, Cell Signaling, #3113)
6. Goat AlexaFluor 594 anti-alpaca (1:400, Jackson Immuno Research, 128-585-232)
7. Donkey AlexaFluor 488 anti-mouse (1:800, Molecular Probes, A212022)
8. Goat AlexaFluor 488 anti-guinea pig (1:800, Molecular Probes, A11073)
9. Donkey AlexaFluor 594 anti-rabbit (1:800, Molecular Probes, A21207)

Validation

1. Nb127D01-ALFA, validation was provided in PMID: 35076390, and in figure S1B.
2. Anti-pH3, validation can be found here: https://www.cellsignal.com/products/primary-antibodies/phospho-histone-h3-ser10-antibody/9701?srsltid=AfmBOooAgAdbcJJNulqr4T3CEJMipRdKWjsbwBnSJ0FVyhiHD7boTtao
3. Anti-p4E-BP, validation can be found here: https://www.cellsignal.com/products/primary-antibodies/phospho-4e-bp1-thr37-46-236b4-rabbit-mab/2855?srsltid=AfmBOorZNjtc4ZK6sLwJKgAoKDBHPR1xssIZUVR007PC5cYk07Epn3aT
4. Anti-dHnf4, validation can be found here: PMID: 30554999, PMID: 19254568.
5. Rabbit Monoclonal Antibody HNF-4 alpha, validation here: https://www.cellsignal.com/products/primary-antibodies/hnf-4-alpha-c11f12-rabbit-monoclonal-antibody/3113
6. AlexaFluor 594 anti-alpaca, validation here: https://www.jacksonimmuno.com/catalog/products/128-585-232
7. AlexaFluor 488 anti-mouse, validation here: https://www.thermofisher.com/antibody/product/Donkey-anti-Mouse-IgG-H-L-Highly-Cross-Adsorbed-Secondary-Antibody-Polyclonal/A-21202
8. AlexaFluor 488 anti-guinea pig, validation here: https://www.thermofisher.com/antibody/product/Goat-anti-Guinea-Pig-IgG-H-L-Highly-Cross-Adsorbed-Secondary-Antibody-Polyclonal/A-11073
9. AlexaFluor 594 anti-rabbit, validation here: https://www.thermofisher.com/antibody/product/Donkey-anti-Rabbit-IgG-H-L-Highly-Cross-Adsorbed-Secondary-Antibody-Polyclonal/A-21207

## Eukaryotic cell lines

Policy information about cell lines and Sex and Gender in Research

| | |
|---|---|
| Cell line source(s) | HepG2 cell line was used, obtained from ATCC. |
| Authentication | Cells obtained from ATCC were not authenticated. |
| Mycoplasma contamination | Not tested for mycoplasma. |
| Commonly misidentified lines (See ICLAC register) | N/A |

## Animals and other research organisms

Policy information about studies involving animals; ARRIVE guidelines recommended for reporting animal research, and Sex and Gender in Research

Laboratory animals

For Drosophila studies: Esgts (esg-Gal4, UAS-GFP, tub-GAL80TS), Esg-LexA and LexAop-yki3SA-GFP, Attp40 UAS-Luciferase are from the Perrimon lab stock collection. The following strains were obtained from the Bloomington Drosophila Stock Center (BL), Vienna Drosophila Resource Center (V) and flyORF (F): UAS-Hnf4 RNAi (BL# 29375), UAS-mElo RNAi (BL# 44510), UAS-yki3SA (BL#28817), UAS-Pvf1-3xHA (F002862), UAS-PvR[A] (BL#58496), UAS-Pvf1 RNAi (V#102699), UAS-PvR RNAi (V#43459), UAS-Luciferase RNAi (BL#31603), Oets (Desat1-Gal4, tubP-Gal80) (BL# 65406), UAS-LpR1 RNAi (BL# 50737), UAS-LpR2 RNAi (BL# 54461). UAS-Hnf4 is a gift from Dr. Carl Thummel; UAS-TSC1, TSC2 is a gift from Dr. Christen K Mirth and UAS-PRAS40 was a gift from Dr. Aurelio Teleman, BtlTS (Btl-Gal4, UAS-srcGFP) and QF6 >QUAS-mtdTomato was a kind gift from Dr. Chrysoula Pitsouli. PromEGS(PromE-GeneSwitch-Gal4) was a gift from Dr. Heinrich Jasper. UAS-CG18609 and mFas-Gal4 were gifts from Dr. Henry Chung. UAS-PRAS40 is a gift from Dr. Aurelio A Teleman.
For mice studies: KrasG12D/+;Lkb1f/f mice, previously described [7, 96], were backcrossed to the FVB strain. Male mice were maintained on a 12-hour light/dark cycle at an ambient temperature of 22°C, with ad libitum access to rodent chow (PicoLab Rodent 5053; Lab Diet, 3.43 kcal/g) and drinking water. Tumors were induced in adult male and female mice (12 to 20 weeks old) via intranasal administration of 75 µL PBS containing $2.5 \times 10^7$ pfu of adenovirus SMV-CRE (Ad5CMV-Cre) purchased from the University of Iowa Gene Transfer Vector Core (Iowa City, IA). The endpoint cutoff for these experiments was 25% weight loss. These experiments used an orthotopic lung cancer model, and thus there was no specific tumor size endpoint.

Wild animals

N/A

Reporting on sex

Lifespan extending effect of mElo knockdown applies to both sexes. Most of experiments were conducted in female flies due to their larger size and thus easier for oenocyte isolation.
HepG2 cell line was isolated from white male.
Male mice liver samples were used in this study.

| | |
|---|---|
| Field-collected samples | N/A |
| Ethics oversight | All animal experiments were conducted with the approval of the Institutional Animal Care and Use Committee (IACUC) at Weill Cornell Medicine (WCM) under protocol number 2013-0116. |

Note that full information on the approval of the study protocol must also be provided in the manuscript.

## Plants

| | |
|---|---|
| Seed stocks | N/A |
| Novel plant genotypes | N/A |
| Authentication | N/A |
