# Supplementary material for: Lipid metabolism of hepatocyte-like cells supports intestinal tumor growth in Drosophila
Source: Nat Commun. 2026 Jul 3;17:8296. doi: 10.1038/s41467-026-75074-w (PMC13470496; doi:10.1038/s41467-026-75074-w)
Supplement: Supplementary file 2 [file 41467_2026_75074_MOESM11_ESM.pdf]

nature portfolio

Peer Review File

Lipid metabolism of hepatocyte-like cells supports intestinal tumor growth in Drosophila

Corresponding Author: Professor Norbert Perrimon

This file contains all reviewer reports in order by version, followed by all author rebuttals in order by version.

Version 0:

Reviewer comments:

Reviewer #1

(Remarks to the Author)
First of all, I would like to thank the editor and the authors for the opportunity to review this manuscript. In this manuscript, Huang et al. describe an intriguing multi-organ interaction involving gut tumors, hepatocyte-like cells (oenocytes), and the trachea in Drosophila melanogaster. Using a well-established Yorkie (Yki)-driven tumor model, the authors delineate a signaling axis from tumor-derived Pvf1 (a PDGF/VEGF-like factor) to Hnf4 (a transcription factor), subsequently regulating the fatty acid elongase mElo, which promotes tracheogenesis and tumor progression. This study uncovers a systemic mechanism that supports tumor growth through lipid metabolism and inter-organ communication. The inter-organ crosstalk uncovered in this study is of broad interest. In the reviewer's opinion, the manuscript has the potential to be published in Nature Communications, pending revision and clarification of several key points outlined below.

[Major comments]
1. The current manuscript structure intermingles data from both healthy (wild-type) and tumor-bearing (Yki) flies, making it difficult to follow the central tumor-related logic. While it is important to show how the pathway functions under physiological and pathological conditions, the mixing of these two contexts throughout the manuscript compromises readability. The reviewer strongly recommends restructuring the manuscript to prioritize tumor-related data, while clearly separating or distinguishing the discussion of wild-type results.

2. The authors focus primarily on oenocytes, but the fat body is also frequently considered functionally analogous to both the liver and adipose tissue in mammals. Therefore, the exclusive designation of oenocytes as hepatocyte-like cells may present an overly narrow view of Drosophila physiology. This is especially important when discussing disease models related to cachexia or systemic metabolic dysfunction. Overlooking the potential contribution of the fat body may weaken the overall argument. A more balanced consideration of both tissues would enhance the physiological relevance and robustness of the authors' conclusions. The reviewer suggests investigating whether the TORC1–Hnf4–mElo pathway is active in the fat body and whether its activity is influenced by tumor presence. Such experiments would help clarify the systemic relevance of the pathway and justify the focus on oenocytes.

3. Tumor progression is currently assessed mainly by counting pH3-positive cells. However, this marker may not fully reflect tumor mass or levels of tumor-derived factors such as Pvf1. A reduction in proliferation could indicate either true tumor regression or indirect effects mediated by systemic crosstalk. This distinction is critical for interpreting cachexia-like phenotypes. Quantifying tumor size or mass (e.g., via imaging or area measurements) would be essential to support the conclusion that systemic effects, such as tissue wasting, are not simply secondary to tumor burden.

4. Given the central role of Hnf4 in the proposed model, additional data on its tissue-specific expression (e.g., scRNA-seq analyses like that of mElo) and its mRNA levels under tumor versus control conditions would be informative. Such data would reinforce the proposed tumor–Hnf4–mElo axis.

5. The rationale behind the selection of Hnf4 targets is not clearly explained. It is also unclear why Hnf4 specifically affects mElo among the listed target genes, and why mElo knockdown yields stronger rescue of lifespan than Hnf4 knockdown. Additional mechanistic experiments, including potentially rescuing or modulating lipid metabolism (e.g., dietary lipid supplementation), would help resolve these points.

6. The absence of metabolism phenotype upon LpR1-2 knockdown raises the question of knockdown efficiency. The authors should assess whether lipid metabolism is actually perturbed in this setting, to validate their genetic perturbation. Without such validation, conclusions regarding pathway dispensability remain premature.

7. The inclusion of mammalian datasets is appreciated, but the current data are limited and somewhat disconnected from the central conclusions. In particular, the assertion in line 377 is not fully supported by data. It would be helpful to provide metabolic data (e.g., expression of Hnf4 target genes and lipid metabolic profiles) in human/mouse models with elevated Hnf4.

8. The study uses the Drosophila Yki tumor model, but its nature and characteristics are not clearly described in the manuscript. This makes comparisons with KrasG12D/+; Lkb1f/f (KL) mice or HepG2 cells less effective. The authors should reconsider their choice of comparative models and revise their discussion to better align with the biological nature of the Yki model.

[Minor Comments]
1. The title should specify the species (e.g., Drosophila) to avoid confusion and better reflect the model system used.

2. The reference list contains several duplications (e.g., refs 28/29 and 31/34) and lacks citations where needed (e.g., lines 42 and 44). Additionally, the manuscript cites only one paper on tumor-liver interaction (ref. 5), despite a rich body of literature on the topic. The authors should revise and enrich the reference list to improve its scholarly quality.

3. The legends for Figure 1K and 1L appear to be swapped and should be corrected.

4. The authors refer to HepG2 as “hepatocytes,” but this cell line is derived from hepatocellular carcinoma and should be referred to as a hepatoma cell line to more accurately reflect its origin.

Reviewer #2

(Remarks to the Author)
In this paper, Huang et al. detail a novel set of physiological processes wherein gut tumors in Drosophila signal to distant hepatocyte-like cells (oenocytes) via PDGF/VEGF-type signaling to promote tracheogenesis and tumor growth. The study identifies a TORC1-Hnf4-mElo relay in oenocytes as a critical driver of altered lipid metabolism and secretion, which in turn facilitates tracheal expansion and tumor progression. Parts of this signaling network have been described previously, for instance mTOR/Hnf4-regulated steatosis in oenocytes and tracheal expansion as a driver of tumorigenesis, but a role for oenocytes in tumor growth and the whole circuit as a unit is novel and quite interesting. The authors further demonstrate that some aspects of this pathway is conserved in human hepatocytes and a mouse tumor model, lending the paper additional relevance. The paper is for the most part clear, and compelling and concisely presented. With few exceptions the experiments are elegantly designed and the data support the conclusions; indeed in many cases the results are robust and striking. A powerful combination of methods is used, and there are some innovative tools, such as the combined use of Gal4 and LexA to drive the expression of different transgenes in tumors and oenocytes. Several genetic rescue experiments are presented (e.g., Hnf4 overexpression with TSC1/2), which are particularly strong in establishing causality. The weakest link in the proposed siganaling network is the function of secreted lipids on the trachea. The data supporting this link, consisting of lipid receptor knockdowns in trachea, are rather weak (Figure 4M-O), and the transfer of oenocyte lipids to trachea is not directly visualized (admittedly, this would be technically challenging to do). So, I was not 100% convinced that uptake of oenocyte-derived lipids by trachea drives tracheal growth and tumorigenesis, even though this is a logical interpretation of the data provided. Apart from that, the paper is missing some simple experimental controls, and lacks some necessary information about experimental details, as listed below. These minor shortcomings should be straightforward to remedy in revision. Overall this is a technically strong, very interesting paper that should be appreciated by a broad audience; I support its publication.
Specific criticisms:
1. It is critical that the Gal4 drivers used for expression in oenocytes, PromE-Gal4 and PromE-gs, are 100% specific to oenocytes. If PromE-Gal4 or PromE-gs is expressed in any other tissues, this would confound most of the results presented in this study The authors need to provide a more definitive evidence that PromE-Gal4 and PromE-gs are oenocyte specific. A paper is cited as showing this (#14, Chatterjee), but that paper lacks convincing evidence of the driver’s specificity and in fact it cites another paper (Billeter et al. Nature, 2009). To support their conclusions, the authors should provide convincing evidence that Prom-Gal4 is completely oenocyte-specific. This could be published data, if it exists.
2. Some experiments lack control conditions. For instance, in Fig 1D, I, J, and K the Prom>Hnf4-i alone genotype is missing. This is also the case for Figure 3G-Q (missing Prom>Pvf1-i or Prom>Pvr alone genotypes). Considering the results that are shown, these are probably not the most important controls, but these controls should nevertheless be included to meet standards in the field.
3. Apart from the effects on mElo, the gene expression data on Hnf4 activity in the tumor condition is rather weak (Lines 169-173, Fig S1F). The authors seem to ignore this data; please discuss this more clearly – what does it mean about Hnf4 activity?
4. The paper presents many experiments in which mTORC1 activity is suppressed in oenocytes by expressing TSC1/TSC1. However, no data is shown in which mTORC1 is hyperactivated. Such experiments could be informative and the authors are encouraged to include them.

5. On lines 261-262 and Figure 3G-L, please note whether gut tumors were actually formed in the Esg>yki;Pvf-I genotype. If tumors where not formed the significance of these results is ambiguous (i.e. if Pvf is required for ISC tumor growth autonomously, rather than as a signal to oenocytes).
6. The effects of LpR1 KD in trachea (Fig 3M-O) are really rather minor, although a major aspect of the paper's conclusions rests on them. Ideally, more convincing data on the effects of lipid uptake in trachea should be provided. Alternatively the authors could qualify their conclusions about this topic.
7. The precise mechanism by which very long-chain fatty acids (VLCFAs) or wax esters promote tracheogenesis remains speculative. More could be said and done about this.
8. While knockdown of the lipid uptake receptor LpR1 in trachea impaired tracheogenesis, a direct visualization or tracing of lipid movement from oenocytes to trachea was not performed. This leaves the proposed role of oenocyte lipids in some doubt. Ideally, this critical link in the signaling relay proposed by the authors could be supported by additional data. Furthermore, the tracheal function of LpR1 in the context of gut tumors was not tested. Testing this would strengthen this weakest aspect of the paper.
9. Clarifying whether the lipids secreted by oenocytes function as signals, structural components, or energy sources for the trachea would significantly advance the mechanistic model proposed in this paper. While it should not be required to explore this issue experimentally, any pertinent data would be a good addition to this paper.
10. A potential confounding effect of systemic Pvf1 signaling should be considered and discussed. Work also from the Perrimon lab has shown that Pvf1 from muscle can regulate oenocyte lipid metabolism, and documented effects on muscle wasting and renal dysfunction in flies. Thus the oenocyte phenotypes presented here might conceivably result indirectly from a systemic metabolic disruption rather than direct tumor-oenocyte crosstalk. It would be possible to test this by checking if muscle-specific Pvf1 knockdown rescues oenocyte Hnf4 activation, and similar experiments. The authors should at least discuss this possible caveat to their conclusions.
Minor comments
11. The manuscript refers to “wild type” flies and “control” flies interchangeably; consistent terminology would improve clarity.
12. The term “normalized area” is used as a label in many Y axis graphs, but this is uninformative. Please re-label these graphs or describe the meaning of “normalized area” in the figure legends.
13. The supplemental figure legends need more information about how the experiments were performed and what the data shows.
14. More information is needed about how the Nb127D01 signal (Hnf4) was quantified in nuclei (Fig 1EF, 2BC.
15. In Figure 2A, the datapoints are missing. Please display them.
16. Are the effects shown in Figure 2D significant? Please provide a statistical analysis.
17. The data shown in Figure 4H-L are negative results, and can be moved to the supplement.
18. The conclusion on lines 410-412 is an overstatement; lipid uptake by trachea was not assayed.
19. There are some grammatical and spelling mistakes:
Page 7, Line 218: "overexprerssing" should be "overexpressing."
Page 8, Line 208: "that that" (duplicate word).
Page 10, Line 275: “suggests": "suggest" (data is plural).
Page 12, Line 323: "oenocyte-specific" (hyphen needed).
Page 13, Line 360: "oenocyte lipid metabolism is important for trachea growth induced by Yki" – "trachea" might be clearer as "tracheal."
Page 13, Line 368: "measure" should be "measured."
Page 14, Line 408: "Similar to tumors" (plural for consistency)
Page 17, Line 490: "confirms" should be "confirmed."

Reviewer #3

(Remarks to the Author)
In this study, Huang et al. revealed a novel mechanism by which gut tumors in adult Drosophila melanogaster reprogram lipid metabolism in distal hepatocyte-like cells (oenocytes) to promote tracheal development and tumor growth. They identified a signaling axis involving tumor-secreted PDGF/VEGF-like factor (Pvf1), which activates the TORC1-Hnf4 pathway in oenocytes, leading to increased production of very long-chain fatty acids (VLCFAs) and wax esters. These lipids are transported via lipoproteins to support tracheal growth around the tumor, a process analogous to angiogenesis in mammals. The authors demonstrate that reducing Hnf4 or the lipid elongase mElo in oenocytes suppresses tumor growth, tracheogenesis, and cachexia-like phenotypes, extending lifespan. The study highlights a tumor-host interaction where tumors non-autonomously reprogram distal lipid metabolism to support their growth.

Overall, findings in this study are interesting, as they bridge tumor biology, lipid metabolism, and angiogenesis, potentially highlighting cancer signaling mechanisms. While the proposed model is conceptually intriguing, I have some concerns regarding experimental design and interpretation of results which could limit the impact of both the broad and succinct mechanisms described in the manuscript.

Major concerns:
1. The manuscript demonstrates that oenocyte-derived lipids (e.g., PC 36:4, PE, wax esters) are transported via lipophorins to promote tracheal growth. However, the precise mechanism by which these lipids contribute to tracheogenesis remains unclear. It is not established whether they serve as structural components, signaling molecules, metabolic substrates, etc. – outside of a brief mention about these particular lipids potentially used to build membranes.

2. The study suggests that tracheal growth supports tumor progression, presumably by enhancing oxygen or nutrient delivery. However, the authors do not provide any experimental evidence of a causal link. Figure S4E indicates limited

effects on ISC proliferation rates in the intestine, and no other phenotype is presented or tested.

3. The mammalian data (HepG2 cells and tumor-bearing mice) are relatively preliminary compared to the extensive in vivo Drosophila experiments and heavily rely on transcript changes to infer conservation of mechanism. For example: a) The HepG2 experiments measure ELOVL1-7 expression but do not confirm functional outcomes (e.g., lipid production, VEGF-A-induced signaling changes, etc.). b) The mouse data show increased hepatic Hnf4 and Elovl7 expression but lack functional validation (e.g., lipidomic profiling of mouse liver or serum, effects of Elovl7 knockdown, etc.). Thus, this dataset does not address whether VEGF-A signaling or changes in distinct lipid metabolic processes in hepatocytes directly contributes to tumor growth or angiogenesis in mammals, limiting the claims of mechanistic conservation / translation.

4. The manuscript uses inconsistent terminology, such as “abdomen area” and “bloating area”, which confuses the interpretation of cachexia-related measurements. The authors should standardize these terms, clarify their biological significance, and provide detailed methods for their quantification. Is “Bloat rates (%)”a better standard, compared to “Abdomen area”, to evaluate the cachexia phenotype?

Furthermore, these phenotypes are very difficult interpret without detailed knowledge of the Drosophila intestinal tumor models, and images need to be included.

5. The statement that “trachea growth, but not ISC growth, requires lipids generated from oenocytes transported through lipophorins” (lines 318–319) is not fully supported. The role of lipophorins in oenocytes (as opposed to tracheal cells) in modulating tumor phenotypes and tracheal growth is not tested.

6. The introduction references “Yki flies” but does not adequately explain the Yorkie-driven intestinal tumor model or its relevance to human cancers (e.g., YAP/TAZ-driven tumors). A brief description of the model and its biological context in Introduction would improve accessibility for readers unfamiliar with Drosophila cancer models.

7. It has been previously reported that muscle secretion of Pvf1 inhibits lipid synthesis in Drosophila oenocytes and regulates fat accumulation in the fat body by activating the Pi3K/Akt/TOR signaling pathway. This study focuses on how Pvf1 secreted by gut tumors promotes oenocytes lipid synthesis and thereby fuels tumor growth, which is contrary to the mechanism proposed in previous studies. Although the background is different (muscle homeostasis vs. tumor context), the authors do not clearly explain why Pvf1 plays such different roles in these two contexts. Also, since Pvf1 has been shown to regulate lipid accumulation in the fat body, it is important that the authors rule out the possibility that the lipid changes observed in Yki flies originate from alterations in the fat body rather than oenocytes.

Minor concerns:
1. Scale bars in figures lack units in the legends. The authors should indicate the units for each figure to ensure clarity and reproducibility.

2. The lipidomic profiling data in figures use “Normalized area” as the unit (e.g., Figure 1J). This term is ambiguous and should be clarified (e.g., normalized to total protein, internal standards, or sample volume) to ensure accurate interpretation.

3. The Y-axis label in Figure 1J appears to omit “x106” or a similar scaling factor, making the data scale unclear. The authors should verify and correct the axis label.

4. The legends for Figure 1K and 1L are reversed.

5. The method for measuring ovary size is inconsistent between Figure S1K (unit: Relative area) and S1L (unit: ovary size). The authors should standardize the measurement method and units across panels and provide a detailed description in the methods.

6. The figure legend for Figure 4 omits the label of “Fig. 4A”.

7. In Figure 4I-K, the authors compare Esg>Luc-i (control) versus Esg>yki,LpR1-i or Esg>yki,LpR2-i. Why are Esg>yki,Luc-i flies not used as a control to assess the specific effects of LpR1/LpR2 knockdown in the context of intestinal tumors?

Version 1:

Reviewer comments:

Reviewer #1

(Remarks to the Author)
Overall, the authors have made a serious effort to address the reviewers’ comments by adding new quantifications and supplementary data. However, the reorganization of the paper, particularly the repositioning of certain figures and arguments, is still somewhat difficult to follow. While several technical concerns have been adequately handled, important conceptual issues remain.

1. Although oenocytes may show relatively high Hnf4 positivity, the snRNA-seq indicates substantial Hnf4-positive fractions across multiple tissues, and the tumor-associated changes are not uniquely or consistently restricted to oenocytes.

Moreover, the proportion of Hnf4-positive cells does not consistently change under Yki tumor conditions. Given that the manuscript discusses systemic phenotypes such as bloating, ovary size changes, and lifespan modulation, it is important to reconsider whether these organismal outcomes can be primarily attributed to the oenocyte–Hnf4–mElo axis. The current data do not exclude the possibility that changes in other Hnf4-expressing tissues contribute to these systemic phenotypes, and this limitation should be more explicitly acknowledged.
2. The mechanistic basis for the selective and pronounced induction of mElo downstream of Hnf4 remains insufficiently resolved. The response largely provides speculative explanations rather than direct experimental evidence addressing target selectivity. As long as the main claim of this paper relies on this particular axis (related to point 1), obtaining more direct evidence supporting target selectivity (for example, ChIP-based or other regulatory evidence) would strengthen this part.
3. The proposed relay between oenocyte-derived lipids and tracheal remodeling remains the least well-supported part of the model. In particular, knockdown experiments with markedly different effect sizes (e.g., relatively weak versus strong phenotypes) are discussed in parallel and described in similarly strong terms. LpR1 knockdown appears weaker than LpR2 knockdown, with substantial overlap of data points. This may be somewhat overinterpreted and may lead to an overemphasis of the lipid uptake component. Clear differentiation of effect strength and a more proportionate interpretation are warranted.

In summary, although the revision improves certain aspects of the manuscript, key conceptual and mechanistic gaps remain, and the framing of the systemic tumor–oenocyte–trachea circuit would benefit from a more cautious and consistent interpretation. These issues may be addressable through clearer framing, more cautious discussion, and appropriate limitation of claims, without necessarily requiring extensive new experiments.

Reviewer #2

(Remarks to the Author)
The revisions to this mauscript are mostly satisfactory. As before, the paper establishes an interesting set of cross-organ interactions between gut tumors, oenocytes, and trachea, that drives tumor growth. The model is coherent an appealing and supported by abundant genetic data, as well as some good lipidomics data, athough direct demonstrations of ligand (Pvf1) and lipid transport between the organs is not provided. The paper has been conscientiously edited to fix mistakes, and a modest amount of new data are included. As described below, there are still some mistakes in the text that need fixing, and a few overstated conclusions that should be qualified. The limitations of the study are not clearly articulated, and it would be helpful for the authors to do this, since alternative explanations of many of the genetic results are possible. Nevertheless, the authors present extensive and compelling evidence for their tumor-coenocyte-trachea-tumor interaction model, and it should be of interest to many readers. The remaining issues are minor, as listed below:
1. The sentence "...Our results also demonstrate that lipids produced..." (lines 31-32, Abstract) is an overstated conclusion that is not definitively warranted by the data. This statement should be changed to more accurately reflect the data, which show changes in hemolymph lipid constituents and alterations effected by depleting the lipoprotein receptor LpR2 in each of the three organs studies. Lipid transport is not directly assessed.
2. Regarding lipid transport, it is surprising that the lipoprotein lipid transporter, Lpp, is not subjected to testing here. Would it not be quite interesting and informative to suppress cross-organ lipid transport by Lpp knockdown? Please comment.
3. Some of the authors' discussion points as made in the rebuttal seem not to have been added to the paper proper. Please review the manuscripts to ensure that important points of discussion from the rebuttal are incorporated into the revised text. For instance, the points made in response to Reviewer #2 questions 7 and 9, and Reviewer #3 question 7, would be helpful also to mention in the paper's main text.
4. There is some sloppy text in the Results section. For instance what are "Yki oenocytes" (lines 187, 188)? Which cells are referred to on line 279, for Hnf4 levels?
5. The results described on lines 333-335, demonstrating a lack effect of Hnf4 depletion in oenocytes, are presented (Fig S4A), but not explained. As these results are inconsistent with the authors' model (Fig 7o) they should be discussed.
5. Some of the Figure citations are wrong (e.g. lines 204, 280, 281, 284).

Reviewer #3

(Remarks to the Author)
The authors have attempted to address most of the concerns that were highlighted in the original version of the manuscript, with some additional experimentation and substantial changes to the text. In general, the overall findings are intriguing, amalgamating tumor signaling mechanisms, distinct aspects of lipid metabolism, and tracheogenesis/angiogenesis to generally support a model of tumor-host interactions in Drosophila. Mechanistically, data support a signaling module by which tumor-secreted Pvf1 (PDGF/VEGF-like factor) activates the TORC1-Hnf4 pathway in lipid-producing oenocytes, leading to increased production of unique lipid species that are likely needed for tracheogenesis.

The revisions have, to an extent, improved the manuscript. However, I have a few comments which do not preclude publication of the manuscript.

First, there are numerous (too many to list) mistakes in the text/Results Section in which the description of data or a reference to a specific Figure panel does not match the actual data/Figure. This includes references in the text to Fig. S1H-I, Fig. 1H, Fig. S1J, Fig. S1K, Fig. S2M and S2D, Fig. 3G-H, and many more. These mismatches make the manuscript

extremely difficult to read and evaluate. In general, the organization and presentation of the data and concepts inhibit readability.

Secondly, even with the revisions, it is still challenging to conceptualize the Pvf1-dependent signaling axis proposed in the manuscript, especially when considering previous findings. Pvf1 has such diverse effects on adult fly metabolism (both in tumor and non-tumor contexts), and new findings presented here suggest even context-specific opposing effects on lipid metabolism within the same tissues, but it's not clear how a single ligand could achieve these effects (even if the secretion source is different). I think these types of paradigms limit the impact, at least mildly, of the new signaling axis described in the manuscript.

Open Access This Peer Review File is licensed under a Creative Commons Attribution 4.0 International License, which permits use, sharing, adaptation, distribution and reproduction in any medium or format, as long as you give appropriate credit to the original author(s) and the source, provide a link to the Creative Commons license, and indicate if changes were made.
In cases where reviewers are anonymous, credit should be given to 'Anonymous Referee' and the source.
The images or other third party material in this Peer Review File are included in the article's Creative Commons license, unless indicated otherwise in a credit line to the material. If material is not included in the article's Creative Commons license and your intended use is not permitted by statutory regulation or exceeds the permitted use, you will need to obtain permission directly from the copyright holder.
To view a copy of this license, visit https://creativecommons.org/licenses/by/4.0/

Response to the reviewers

Reviewer #1 (Remarks to the Author):

First of all, I would like to thank the editor and the authors for the opportunity to review this manuscript. In this manuscript, Huang et al. describe an intriguing multi-organ interaction involving gut tumors, hepatocyte-like cells (oenocytes), and the trachea in Drosophila melanogaster. Using a well-established Yorkie (Yki)-driven tumor model, the authors delineate a signaling axis from tumor-derived Pvf1 (a PDGF/VEGF-like factor) to Hnf4 (a transcription factor), subsequently regulating the fatty acid elongase mElo, which promotes tracheogenesis and tumor progression. This study uncovers a systemic mechanism that supports tumor growth through lipid metabolism and inter-organ communication. The inter-organ crosstalk uncovered in this study is of broad interest. In the reviewer's opinion, the manuscript has the potential to be published in Nature Communications, pending revision and clarification of several key points outlined below.
We thank the reviewer for the positive comments.
[Major comments]
1. The current manuscript structure intermingles data from both healthy (wild-type) and tumor-bearing (Yki) flies, making it difficult to follow the central tumor-related logic. While it is important to show how the pathway functions under physiological and pathological conditions, the mixing of these two contexts throughout the manuscript compromises readability. The reviewer strongly recommends restructuring the manuscript to prioritize tumor-related data, while clearly separating or distinguishing the discussion of wild-type results.
-In response to the reviewer’s suggestion, we revised Figures 2 and 3 to present the Yki tumor dataset first, improving the clarity and flow of the data presentation.

-We also reordered Figures 5 and 6 to prioritize the Yki dataset, so that the key tumor-related findings are introduced earlier and interpreted in a more coherent sequence.

2. The authors focus primarily on oenocytes, but the fat body is also frequently considered functionally analogous to both the liver and adipose tissue in mammals. Therefore, the exclusive designation of oenocytes as hepatocyte-like cells may present an overly narrow view of Drosophila physiology. This is especially important when discussing disease models related to cachexia or systemic metabolic dysfunction. Overlooking the potential contribution of the fat body may weaken the overall argument. A more balanced consideration of both tissues would enhance the physiological relevance and robustness of the authors’ conclusions. The reviewer suggests investigating whether the TORC1–Hnf4–mElo pathway is active in the fat body and whether its activity is influenced by tumor presence. Such experiments would help clarify the systemic relevance of the pathway and justify the focus on oenocytes.
-We thank the reviewer for the insights. To address this, we included the following data to show the relevance of Hnf4 under tumor context:

1. mElo expression is highly enriched in oenocytes and is strongly upregulated in oenocytes from Yki flies, whereas only minimal changes are observed in the fat body. This is supported by our single-nucleus RNA-seq analysis across multiple tissues from control and Yki flies, in which CG18609 corresponds to mElo.

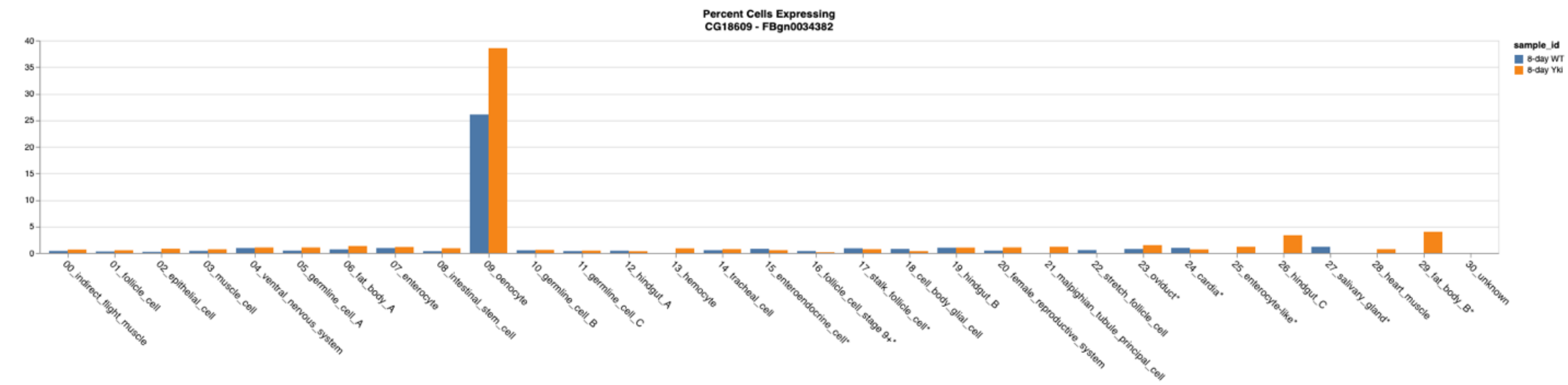

2. Knockdown of Hnf4 and mElo in the fat body did not affect gut tracheal morphology (Figure S6H–M). In contrast, simultaneous reduction of these genes in oenocytes led to a significant decrease in gut tracheal growth (Figure 6E–P). This data was added to the main manuscript (line 378-384).

These data suggest that oenocytes play a more prominent role than the fat body in regulating tracheal growth.

3. Tumor progression is currently assessed mainly by counting pH3-positive cells. However, this marker may not fully reflect tumor mass or levels of tumor-derived factors such as Pvf1. A reduction in proliferation could indicate either true tumor regression or indirect effects mediated by systemic crosstalk. This distinction is critical for interpreting cachexia-like phenotypes. Quantifying tumor size or mass (e.g., via imaging or area measurements) would be essential to support the conclusion that systemic effects, such as tissue wasting, are not simply secondary to tumor burden.

We appreciate the reviewer's suggestion regarding tumor quantification. Accordingly, we quantified tumor burden as the percentage of GFP-positive area across the entire gut, as GFP specifically marks ISCs/tumor cells in our experimental system. In particular, the ISC-specific driver (esg-GAL4, UAS-GFP; tub-GAL80^{TS}) and the Yki activation construct (LexAOP-Yki-GFP) enable direct visualization and quantification of ISC/tumor expansion by measuring the GFP+ region.

New quantification was added in Figure 1SC, Figure 1SJ, Figure S3D, Figure S3F, Figure 4M.

4. Given the central role of Hnf4 in the proposed model, additional data on its tissue-specific expression (e.g., scRNA-seq analyses like that of mElo) and its mRNA levels under tumor versus control conditions would be informative. Such data would reinforce the proposed tumor–Hnf4–mElo axis.

Please see the snRNA-seq analysis of control and Yki flies across multiple tissues. Notably, oenocytes (cluster 9) display the highest proportion of Hnf4-expressing cells. Although Hnf4 is also upregulated in the fat body of Yki flies, its downstream target mElo is markedly more enriched and strongly induced in oenocytes (see our response to Comment 2). Together, these data support oenocytes as the primary tissue in which to investigate the

tumor–Hnf4–mElo regulatory axis.

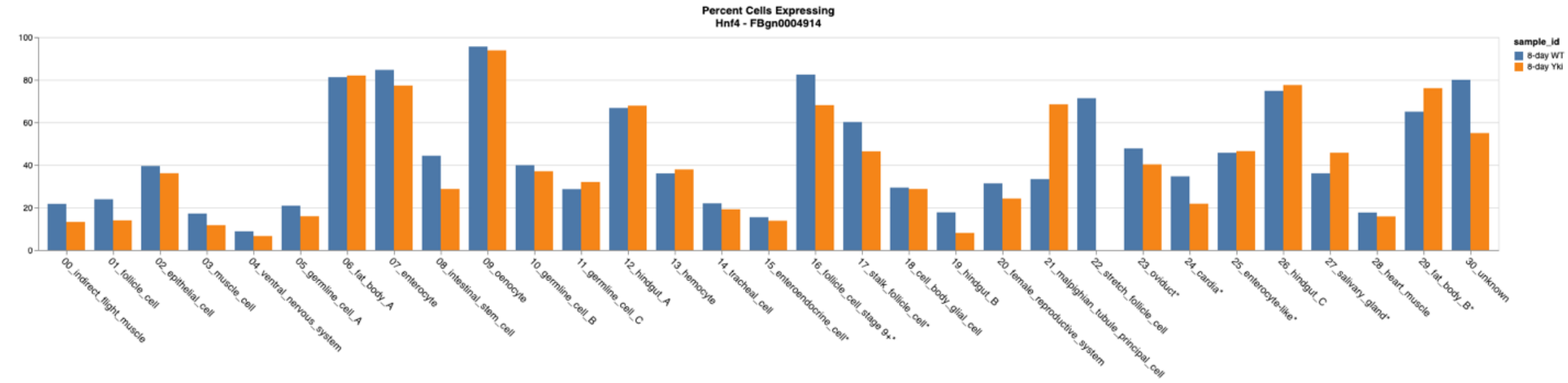

5. The rationale behind the selection of Hnf4 targets is not clearly explained. It is also unclear why Hnf4 specifically affects mElo among the listed target genes, and why mElo knockdown yields stronger rescue of lifespan than Hnf4 knockdown. Additional mechanistic experiments, including potentially rescuing or modulating lipid metabolism (e.g., dietary lipid supplementation), would help resolve these points.

We thank the reviewer for the comment.

- We selected mElo because it is robustly induced in oenocytes from tumor-bearing flies, as identified in our mini-PCR screen (Figure 1J and Figure S1G). We further propose that the tumor metabolic environment may reprogram Hnf4-dependent transcription, potentially through changes in (i) Hnf4 cofactor usage and/or (ii) ligand availability, thereby favoring induction of specific downstream targets such as mElo. Consistent with this idea, prior studies have shown that Hnf4α transcriptional output can shift depending on its interacting partners—for example, Hnf4α can cooperate with ChREBP to promote lipogenic gene expression such as Fasn during feeding [1, 2]. whereas under fasting conditions Hnf4α can associate with Foxo1 to enhance transcription of gluconeogenic genes such as G6Pase [3]. In addition, Hnf4α activity can be modulated by long-chain fatty acids [4], raising the possibility that altered lipid availability in tumor-bearing flies (e.g., from TAG mobilization) may influence Hnf4 activity and target selection. Together, these observations support a model in which tumor-associated changes in Hnf4 partners and/or lipid ligands promote a shift in Hnf4 transcriptional programs in Yki oenocytes, contributing to strong mElo induction. We also added this to the discussion section (Line 496-510).

- In response to "why mElo knockdown yields stronger rescue of lifespan than Hnf4 knockdown", we checked the expression of *mElo* in Esg-LexA;PromE-Gal4> LexAOP-Yki ; UAS-Hnf4 RNAi, comparing that with Esg-LexA;PromE-Gal4> LexAOP-Yki ; UAS-mElo RNAi. mElo's knockdown efficiency is better than Hnf4 RNAi, thus yielding greater rescue effects in the tumor flies.

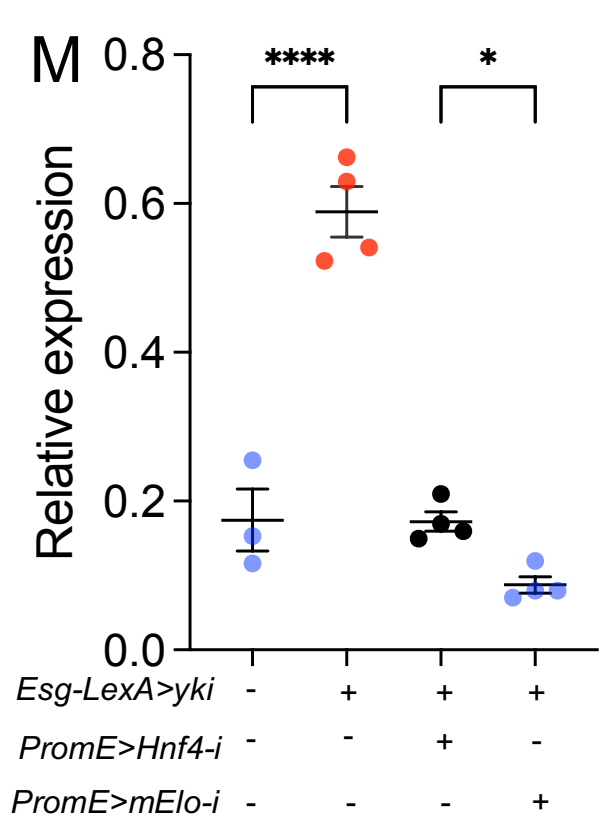

6. The absence of metabolism phenotype upon LpR1-2 knockdown raises the question of knockdown efficiency. The authors should assess whether lipid metabolism is actually perturbed in this setting, to validate their genetic perturbation. Without such validation, conclusions regarding pathway dispensability remain premature.
We checked the knockdown efficiency of both LpR1-2 knockdown lines, both RNAi lines provide high knockdown efficiency (Figure S4F-G):

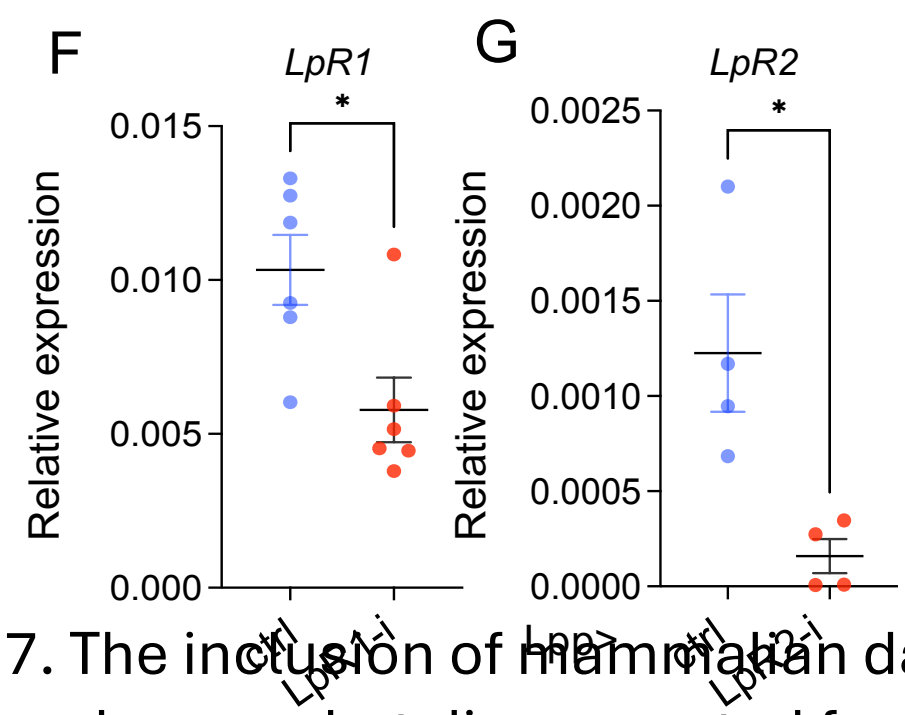

7. The inclusion of mammalian datasets is appreciated, but the current data are limited and somewhat disconnected from the central conclusions. In particular, the assertion in line 377 is not fully supported by data. It would be helpful to provide metabolic data (e.g., expression of Hnf4 target genes and lipid metabolic profiles) in human/**mouse** models with elevated Hnf4.
-We measured Hnf4α target gene, *ApoB*, in human HepG2 cells with VEGF-A (Figure 7F), which showed higher expression under VEGF-A treatment. Consistently, we observed an increased level of HNA4 α localization in the HepG2 cells treated with VEGF-A (Figure S7A-B). We also found the induction of Hnf4 α target genes *Acc1* and *Fasn* in the liver samples of KL mice (Figure 7J-L).
-We performed lipidomic analysis in conditioned medium from VEGF-A (Figure 7G) treated HepG2 cells, and we observed a robust increase in WE (Figure 7G), like that seen in Yki flies (Figure 1A-C).
-Consistent with the expression data, from lipidomic profiling of serum samples with tumor bearing mice and control mice, it revealed higher level of WE in the tumor mice serum (Figure 7M), which are mostly constituted with very long chain fatty acids (Figure S7C). The lipidomic analysis also showed that out of 147 significantly changed lipid species, most of them (118) are induced and only 29 of lipids are reduced (Figure S7C). From the summed lipid species, we found that although the phosphatidylcholine (PC) and triglycerides (TG) are reduced in tumor bearing mice serum, majority of other lipid species, such as Acylcarnitine (AcCa), lysophosphatidylcholine (LPC) are highly induced in tumor bearing mice (Figure 7N).

8. The study uses the *Drosophila* Yki tumor model, but its nature and characteristics are not clearly described in the manuscript. This makes comparisons with KrasG12D/+;

Lkb1f/f (KL) mice or HepG2 cells less effective. The authors should reconsider their choice of comparative models and revise their discussion to better align with the biological nature of the Yki model.
We thank the reviewer for the constructive comments. We respectfully suggest that the Yki fly tumor model and the KL mouse model are comparable in the context of our study for the following reasons:
Shared cachexia-like systemic responses and conserved secreted signaling. Both Yki tumors in flies and KL tumors in mice elicit robust cachexia-associated phenotypes driven by conserved tumor-derived factors. In flies, Yki tumors induce Upd3 (the functional ortholog of mammalian IL-6) as well as Pvf1 (a PDGF/VEGF-related factor). Notably, IL-6- and PDGF/VEGF-associated inflammatory programs are also commonly elevated in KRAS-driven lung cancer models [5-8]. Therefore, comparing downstream signaling (including Pvf1-associated pathways) across the Yki and KL systems is biologically justified.
Similar mode and timing of tumor initiation. In the KL mouse model, lung tumors are initiated in adult animals by intranasal delivery of Cre-expressing adenovirus, rather than arising developmentally. Likewise, in our fly system, Yki-driven tumor induction is initiated in adult flies, enabling comparison of systemic metabolic responses to tumors that arise after normal development.

Prior evidence supporting cross-species mechanistic conservation. In our recent work, we used the Yki fly tumor model to uncover a conserved role of IL-6–like signaling in promoting gluconeogenesis that contributes to cancer cachexia, and we validated this mechanism in the KL mouse model [9]. This precedent underscores the value of directly comparing these two experimental systems.

[Minor Comments]
1. The title should specify the species (e.g., Drosophila) to avoid confusion and better reflect the model system used.
We thank the reviewer's comments. We changed the title to: Lipid metabolism of hepatocyte-like cells supports intestinal tumor growth in *Drosophila*.

2. The reference list contains several duplications (e.g., refs 28/29 and 31/34) and lacks citations where needed (e.g., lines 42 and 44). Additionally, the manuscript cites only one paper on tumor-liver interaction (ref. 5), despite a rich body of literature on the topic. The authors should revise and enrich the reference list to improve its scholarly quality.
We thank the reviewer for the thoughtful comment, we have modified the citations accordingly (line 44 and line 56).

3. The legends for Figure 1K and 1L appear to be swapped and should be corrected.
See new figure 1K and 1L legend.
4. The authors refer to HepG2 as "hepatocytes," but this cell line is derived from hepatocellular carcinoma and should be referred to as a hepatoma cell line to more accurately reflect its origin.

We modified the manuscript accordingly to avoid referring HepG2 as “hepatocytes”.

Reviewer #2 (Remarks to the Author):

In this paper, Huang et al. detail a novel set of physiological processes wherein gut tumors in Drosophila signal to distant hepatocyte-like cells (oenocytes) via PDGF/VEGF-type signaling to promote tracheogenesis and tumor growth. The study identifies a TORC1-Hnf4-mElo relay in oenocytes as a critical driver of altered lipid metabolism and secretion, which in turn facilitates tracheal expansion and tumor progression. Parts of this signaling network have been described previously, for instance mTOR/Hnf4-regulated steatosis in oenocytes and tracheal expansion as a driver of tumorigenesis, but a role for oenocytes in tumor growth and the whole circuit as a unit is novel and quite interesting. The authors further demonstrate that some aspects of this pathway is conserved in human hepatocytes and a mouse tumor model, lending the paper additional relevance. The paper is for the most part clear, and compelling and concisely presented. With few exceptions the experiments are elegantly designed and the data support the conclusions; indeed in many cases the results are robust and striking. A powerful combination of methods is used, and there are some innovative tools, such as the combined use of Gal4 and LexA to drive the expression of different transgenes in tumors and oenocytes. Several genetic rescue experiments are presented (e.g., Hnf4 overexpression with TSC1/2), which are particularly strong in establishing causality. The weakest link in the proposed siganaling network is the function of secreted lipids on the trachea. The data supporting this link, consisting of lipid receptor knockdowns in trachea, are rather weak (Figure 4M-O), and the transfer of oenocyte lipids to trachea is not directly visualized (admittedly, this would be technically challenging to do). So, I was not 100% convinced that uptake of oenocyte-derived lipids by trachea drives tracheal growth and tumorigenesis, even though this is a logical interpretation of the data provided. Apart from that, the paper is missing some simple experimental controls, and lacks some necessary information about experimental details, as listed below. These minor shortcomings should be straightforward to remedy in revision. Overall this is a technically strong, very interesting paper that should be appreciated by a broad audience; I support its publication.
We thank the reviewer for the positive comments.
Specific criticisms:
1. It is critical that the Gal4 drivers used for expression in oenocytes, PromE-Gal4 and PromE-gs, are 100% specific to oenocytes. If PromE-Gal4 or PromE-gs is expressed in any other tissues, this would confound most of the results presented in this study. The authors need to provide a more definitive evidence that PromE-Gal4 and PromE-gs are oenocyte specific. A paper is cited as showing this (#14, Chatterjee), but that paper lacks convincing evidence of the driver’s specificity and in fact it cites another paper (Billeter et al. Nature, 2009). To support their conclusions, the authors should provide convincing evidence that Prom-Gal4 is completely oenocyte-specific. This could be published data, if it exists.

It was previously published that PromE-Gal4 is highly specific in female oenocytes [10, 11]. In experiment we conducted using males (PromE-Gal4 > mElo RNAi). However, mElo is not expressed in the ejaculatory bulb in male flies, suggesting that the effect is specific to oenocytes.

J

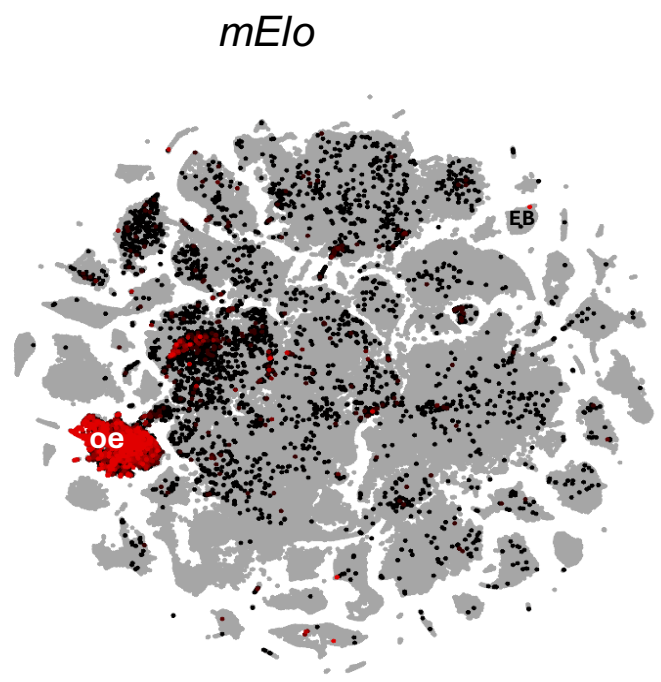

Red marks the level of mElo expression in oenocyte cluster and ejaculatory bulb.

2. Some experiments lack critical controls. For instance, in Fig 1D, I, J, and K the Prom>Hnf4-i alone genotype is missing. This is also the case for Figure 3G-Q (missing Prom>Pvf1-i or Prom>Pvr alone genotypes). Considering the results that are shown, these are probably not the most important controls, but these controls should nevertheless be included to meet standards in the field.
We thank the reviewer for this constructive comment, we have added the control as requested in the main figures. See Figure 1D, 1G, 1H, 1I, 1K, 2A, 2D-E, 3A-H, Figure S3C-E, Figure 4H-Q.

3. Apart from the effects on mElo, the gene expression data on Hnf4 activity in the tumor condition is rather weak (Lines 169-173, Fig S1F). The authors seem to ignore this data; please discuss this more clearly – what does it mean about Hnf4 activity?
We thank the reviewer for the comment.
- We selected mElo because it is robustly induced in oenocytes from tumor-bearing flies, as identified in our mini-PCR screen (Figure 1J and Figure S1G). We further propose that the tumor metabolic environment may reprogram Hnf4-dependent transcription, potentially through changes in (i) Hnf4 cofactor usage and/or (ii) ligand availability, thereby favoring induction of specific downstream targets such as mElo. Consistent with this idea, prior studies have shown that Hnf4α transcriptional output can shift depending on its interacting partners—for example, Hnf4α can cooperate with ChREBP to promote lipogenic gene expression such as Fasn during feeding [1, 2]. whereas under fasting conditions Hnf4α can associate with Foxo1 to enhance transcription of gluconeogenic genes such as G6Pase [3]. In addition, Hnf4α activity can be modulated by long-chain fatty acids [4], raising the possibility that altered lipid availability in tumor-bearing flies (e.g., from TAG mobilization) may influence Hnf4 activity and target selection. Together, these observations support a model in which tumor-associated changes in Hnf4 partners and/or lipid ligands promote a

shift in Hnf4 transcriptional programs in Yki oenocytes, contributing to strong mElo induction. We also added this to the discussion section (Line 496-510).

4. The paper presents many experiments in which mTORC1 activity is suppressed in oenocytes by expressing TSC1/TSC1. However, no data is shown in which mTORC1 is hyperactivated. Such experiments could be informative and the authors are encouraged to include them.

We have shown increased level of P-4EBP (a measurement of mTORC1 activity) in the Yki flies' oenocytes (Figure 2B-C).

5. On lines 261-262 and Figure 3G-L, please note whether gut tumors were actually formed in the Esg>yki;Pvf-I genotype. If tumors where not formed the significance of these results is ambiguous (i.e. if Pvf is required for ISC tumor growth autonomously, rather than as a signal to oenocytes).

It was shown in previous literature that there is still tumor formation in Esg>yki;Pvf-I flies [12]. We also discovered that there is tumor growth in Esg>yki;Pvf-I flies measured by % of GFP+ region (Figure S3D).

6. The effects of LpR1 KD in trachea (Fig 3M-O) are really rather minor, although a major aspect of the paper's conclusions rests on them. Ideally, more convincing data on the effects of lipid uptake in trachea should be provided. Alternatively the authors could qualify their conclusions about this topic.

Even though LpR1 KD in trachea has dramatic effect, we found the knockdown of LpR1 in trachea has minimal effect on tumor growth. Because this paper primarily focuses on the effect of lipids on tumor growth, we decided to remove the LpR1 KD data in trachea under normal physiological condition.

7. The precise mechanism by which very long-chain fatty acids (VLCFAs) or wax esters promote tracheogenesis remains speculative. More could be said and done about this.

Lipid elevation could promote tracheogenesis by increasing reactive oxygen species (ROS), which has been implicated as a driver of tracheal growth [13]. WEs can be hydrolyzed into fatty acids and fatty alcohols [14]. VLCFAs released from WEs may undergo peroxisomal oxidation, generating H_2O_2 as a byproduct, which can stimulate tracheogenesis [13]. In parallel, fatty acids can be transported to mitochondria for β-oxidation, and high fatty-acid flux through mitochondrial respiration can increase electron leak from the ETC and elevate ROS production, potentially contributing to tracheogenesis [15].

8. While knockdown of the lipid uptake receptor LpR1 in trachea impaired tracheogenesis, a direct visualization or tracing of lipid movement from oenocytes to trachea was not performed. This leaves the proposed role of oenocyte lipids in some doubt. Ideally, this critical link in the signaling relay proposed by the authors could be supported by additional data. Furthermore, the tracheal function of LpR1 in the context of gut tumors was not tested. Testing this would strengthen this weakest aspect of the paper.

We thank the reviewers for this critical comment. It has been reported that restricting tracheal growth delays tumor development [35]. Consistently, knocking down LpR2, but

not LpR1, using a temperature sensitive trachea-specific driver (btl-GAL4, UAS-srcGFP, Tub-GAL80TS, referred to as Btl) in the Yki flies prevented Yki-induced ISC proliferation (Figure 4L) and the tumor burden (Figure 4M). Consistently, it also reduced the level of bloating (Figure 4N), tracheal number of branches, tube area and skeleton length compared to control flies (Btl>+) (Figure 4O-Q). Knocking down LpR2 in the normal condition also reduced tracheal branch number skeleton length and total tube area (Figure 4O-Q).

Interestingly, we found that oenocyte-specific depletion of LpR2 also altered gut tracheal morphology under basal conditions (Figure S4I–K), reflecting that the trafficking of phospholipids, WEs and AcCas from oenocytes relies on the uptake of LPPs. Altogether, these findings are consistent with a model in which tracheal morphology, rather than ISC proliferation, depends on lipids generated or processed in oenocytes and delivered via circulating LPP particles.

9. Clarifying whether the lipids secreted by oenocytes function as signals, structural components, or energy sources for the trachea would significantly advance the mechanistic model proposed in this paper. While it should not be required to explore this issue experimentally, any pertinent data would be a good addition to this paper.

We thank the reviewer for the constructive feedback, and we have added the following to the discussion section. Our data indicate that wax esters (WEs) and acylcarnitines (AcCas) are elevated in tumor-bearing flies and mice (Figure 1A and 7N). Notably, in both models we observe an increase in WEs containing very-long-chain fatty acids (VLCFAs), consistent with the elevated expression of Hnf4 and fatty-acid elongases in the KL mouse liver as well as in oenocytes from Yki flies. Functionally, increased AcCas and WEs could contribute to tracheogenesis by supporting local energetic demand. For example, hepatocyte-derived plasma acylcarnitines have been shown to be required for systemic thermogenesis under cold exposure in mice, where peripheral tissues such as brown adipose tissue, skeletal muscle, and heart take up circulating acylcarnitines and oxidize them to fuel metabolism [16]. In addition, wax esters can serve as high-density neutral lipid reserves that are mobilized to meet energetic demands in diverse physiological contexts (e.g., diapause and reproduction in marine organisms). Alternatively, lipid elevation could promote tracheogenesis by increasing reactive oxygen species (ROS), which has been implicated as a driver of tracheal growth [13]. WEs can be hydrolyzed into fatty acids and fatty alcohols [14]. VLCFAs released from WEs may undergo peroxisomal oxidation, generating H_2O_2 as a byproduct, which can stimulate tracheogenesis [13]. In parallel, fatty acids can be transported to mitochondria for β-oxidation, and high fatty-acid flux through mitochondrial respiration can increase electron leak from the ETC and elevate ROS production, potentially contributing to tracheogenesis [15]. These studies also leave open the possibility that metabolites derived from these lipid species act as signaling molecules that promote tracheogenesis.

10. A potential confounding effect of systemic Pvf1 signaling should be considered and discussed. Work also from the Perrimon lab has shown that Pvf1 from muscle can regulate oenocyte lipid metabolism, and documented effects on muscle wasting and renal dysfunction in flies. Thus the oenocyte phenotypes presented here might conceivably

result indirectly from a systemic metabolic disruption rather than direct tumor-oenocyte crosstalk. It would be possible to test this by checking if muscle-specific Pvf1 knockdown rescues oenocyte Hnf4 activation, and similar experiments. The authors should at least discuss this possible caveat to their conclusions.

We thank the reviewer for this comment.

In the Yki context, pvf1 in muscle is not significantly elevated (Figure S3), thus rendering its effects under Yki minimal.

Minor comments

11. The manuscript refers to "wild type" flies and "control" flies interchangeably; consistent terminology would improve clarity.

We changed all "wild type" to "control".

12. The term "normalized area" is used as a label in many Y axis graphs, but this is uninformative. Please re-label these graphs or describe the meaning of "normalized area" in the figure legends.

We changed "normalized area" to "normalized intensity". Normalized intensity was calculated as the integrated LC–MS peak area for each lipid species, normalized to the sample's total signal (total reads) and to the analyzed sample volume.

13. The supplemental figure legends need more information about how the experiments were performed and what the data shows.

We added more details to the figure legends as suggested.

14. More information is needed about how the Nb127D01 signal (Hnf4) was quantified in nuclei (Fig 1EF, 2BC.

We added the following quantification method to the Method section:

Quantification of nucleus Hnf4 intensity was conducted using ImageJ. Background subtraction was performed using the rolling ball radius at 50 pixels. We apply threshold to the DAPI channel and using analyze particles to select nucleus as Region Of Interest (ROI), size: 10-2000; circularity: 0.1-1. Mean gray value was measured for each ROI.

15. In Figure 2A, the datapoints are missing. Please display them.

We have modified accordingly, adding significance testing as well.

16. Are the effects shown in Figure 2D significant? Please provide a statistical analysis.

The data presented was the module score, and its primary function is to directly visualize the expression of a gene module within single cell. A positive score indicates higher-than-average expression of the gene set compared to background. However, significance test is not regularly conducted for this analysis between different samples.

17. The data shown in Figure 4H-L are negative results, and can be moved to the supplement.

We removed Figure 4H from the main figure, but we believe figure 4I-K present important finding of the paper, that is lipid transport is not required in the ISC for its growth and trachea induction.

18. The conclusion on lines 410-412 is an overstatement; lipid uptake by trachea was not assayed.

We modified the sentence to the following: “Lipids from oenocytes are possibly acquired by the tracheal cells instead of ISCs, as knockdowns of *LpR1* and *LpR2* in ISCs have no effects in tumor growth”

19. There are some grammatical and spelling mistakes:

We thank the reviewer for the detailed suggestions, we modified accordingly.

Page 7, Line 218: "overexprerssing" should be "overexpressing."
Page 8, Line 208: "that that" (duplicate word).
Page 10, Line 275: “suggests": "suggest" (data is plural).
Page 12, Line 323: "oenocyte-specific" (hyphen needed).
Page 13, Line 360: "oenocyte lipid metabolism is important for trachea growth induced by Yki" – "trachea" might be clearer as "tracheal."
Page 13, Line 368: "measure" should be "measured."
Page 14, Line 408: "Similar to tumors" (plural for consistency)
Page 17, Line 490: "confirms" should be "confirmed."

Reviewer #3 (Remarks to the Author):

In this study, Huang et al. revealed a novel mechanism by which gut tumors in adult Drosophila melanogaster reprogram lipid metabolism in distal hepatocyte-like cells (oenocytes) to promote tracheal development and tumor growth. They identified a signaling axis involving tumor-secreted PDGF/VEGF-like factor (Pvf1), which activates the TORC1-Hnf4 pathway in oenocytes, leading to increased production of very long-chain fatty acids (VLCFAs) and wax esters. These lipids are transported via lipoproteins to support tracheal growth around the tumor, a process analogous to angiogenesis in mammals. The authors demonstrate that reducing Hnf4 or the lipid elongase mElo in oenocytes suppresses tumor growth, tracheogenesis, and cachexia-like phenotypes, extending lifespan. The study highlights a tumor-host interaction where tumors non-autonomously reprogram distal lipid metabolism to support their growth.

Overall, findings in this study are interesting, as they bridge tumor biology, lipid metabolism, and angiogenesis, potentially highlighting cancer signaling mechanisms. While the proposed model is conceptually intriguing, I have some concerns regarding experimental design and interpretation of results which could limit the impact of both the broad and succinct mechanisms described in the manuscript.

We appreciate the positive feedback from the reviewers!

Major concerns:

1. The manuscript demonstrates that oenocyte-derived lipids (e.g., PC 36:4, PE, wax esters) are transported via lipophorins to promote tracheal growth. However, the precise mechanism by which these lipids contribute to tracheogenesis remains unclear. It is not established whether they serve as structural components, signaling molecules, metabolic substrates, etc. – outside of a brief mention about these particular lipids potentially used to build membranes.

We thank the reviewer for the constructive feedback, and we have added the following to the discussion section. Our data indicate that wax esters (WEs) and acylcarnitines (AcCas) are elevated in tumor-bearing flies and mice (Figure 1A and 7N). Notably, in both models we observe an increase in WEs containing very-long-chain fatty acids (VLCFAs), consistent with the elevated expression of Hnf4 and fatty-acid elongases in the KL mouse liver as well as in oenocytes from Yki flies. Functionally, increased AcCas and WEs could contribute to tracheogenesis by supporting local energetic demand. For example, hepatocyte-derived plasma acylcarnitines have been shown to be required for systemic thermogenesis under cold exposure in mice, where peripheral tissues such as brown adipose tissue, skeletal muscle, and heart take up circulating acylcarnitines and oxidize them to fuel metabolism [16]. In addition, wax esters can serve as high-density neutral lipid reserves that are mobilized to meet energetic demands in diverse physiological contexts (e.g., diapause and reproduction in marine organisms). Alternatively, lipid elevation could promote tracheogenesis by increasing reactive oxygen species (ROS), which has been implicated as a driver of tracheal growth [13]. WEs can be hydrolyzed into fatty acids and fatty alcohols [14]. VLCFAs released from WEs may undergo peroxisomal oxidation, generating H_2O_2 as a byproduct, which can stimulate tracheogenesis [13]. In parallel, fatty acids can be transported to mitochondria for β-oxidation, and high fatty-acid flux through mitochondrial respiration can increase electron leak from the ETC and elevate ROS production, potentially contributing to tracheogenesis [15]. These studies also leave open the possibility that metabolites derived from these lipid species act as signaling molecules that promote tracheogenesis.

2. The study suggests that tracheal growth supports tumor progression, presumably by enhancing oxygen or nutrient delivery. However, the authors do not provide any experimental evidence of a causal link. Figure S4E indicates limited effects on ISC proliferation rates in the intestine, and no other phenotype is presented or tested.
-It has been previously published that inhibition of tracheal growth stops tumor proliferation and extends the lifespan of tumor bearing flies [13]. We added experimental data to support this link. We found that reducing lipoprotein receptor 2 (*LpR2)* in trachea specifically blocked tumor proliferation, reduced the level of tumor induced cachexia and tumor induced tracheal growth (Figure 4L-Q).

3. The mammalian data (HepG2 cells and tumor-bearing mice) are relatively preliminary compared to the extensive in vivo Drosophila experiments and heavily rely on transcript changes to infer conservation of mechanism. For example: a) The HepG2 experiments measure ELOVL1-7 expression but do not confirm functional outcomes (e.g., lipid production, VEGF-A-induced signaling changes, etc.). b) The mouse data show increased hepatic Hnf4 and Elovl7 expression but lack functional validation (e.g., **lipidomic profiling of mouse liver or serum**, effects of Elovl7 knockdown, etc.). Thus, this dataset does not address whether VEGF-A signaling or changes in distinct lipid metabolic processes in hepatocytes directly contributes to tumor growth or angiogenesis in mammals, limiting the claims of mechanistic conservation / translation.

We thank the reviewer for this important suggestion. In the HepG2 experiments, we assessed an HNF4α target gene and found that VEGF-A treatment significantly increased expression of APOB. In parallel, lipidomic profiling of the HepG2 culture medium following VEGF-A addition revealed a robust increase in wax esters (WE) (Figure 7G). Consistent with these transcriptional and metabolic changes, immunostaining showed enhanced nuclear localization of HNF4α in HepG2 cells upon VEGF-A treatment.

To extend these findings in vivo, we will quantify Hnf4 target genes in liver samples (e.g., Acc1 and Fasn). Notably, we observed increased expression of these genes in KL mouse livers, consistent with elevated HNF4α activity. Serum lipidomics from KL mice further showed higher levels of multiple lipid classes, including WE, acylcarnitines (AcCa), LPE, MG, and MGMG (Figure 7M–7N). Importantly, many of the induced WE species contained very-long-chain fatty acids (VLCFAs) (Figure S7C), consistent with elevated Elovl7 expression in KL mouse liver. Overall, among 147 significantly altered lipid species, the majority (118) were increased whereas 29 were decreased (Figure S7C). Together, these data support a conserved response in which VEGF-A signaling reprograms hepatocyte lipid metabolism, which may contribute to tumor growth and tumor-associated metabolic remodeling in mammals.

4. The manuscript uses inconsistent terminology, such as “abdomen area” and “bloating area”, which confuses the interpretation of cachexia-related measurements. The authors should standardize these terms, clarify their biological significance, and provide detailed methods for their quantification. Is “Bloat rates (%)”a better standard, compared to “Abdomen area”, to evaluate the cachexia phenotype?
Furthermore, these phenotypes are very difficult interpret without detailed knowledge of the Drosophila intestinal tumor models, and images need to be included.
-We thank the reviewer for the critical comment. Abdominal bloating is caused by fluid accumulation from tissue wasting in flies. We clarified this in the manuscript and corrected the misuse of terms and included pictures to better illustrate the “Abdomen area” (Figure S1D).

5. The statement that “trachea growth, but not ISC growth, requires lipids generated from oenocytes transported through lipophorins” (lines 318–319) is not fully supported. The role of lipophorins in oenocytes (as opposed to tracheal cells) in modulating tumor phenotypes and tracheal growth is not tested.
-We depleted *LpR2* specifically in oenocytes and observed reduced tracheal growth under normal conditions (Figure S4I-K), suggesting that oenocyte-derived lipids directly regulate tracheal growth.

6. The introduction references “Yki flies” but does not adequately explain the Yorkie-driven intestinal tumor model or its relevance to human cancers (e.g., YAP/TAZ-driven tumors). A brief description of the model and its biological context in Introduction would improve accessibility for readers unfamiliar with *Drosophila* cancer models.

-We added a description for Yki flies and its relevance to human cancer in the manuscript: "The adult Drosophila midgut is a powerful model for investigating tumorigenesis and tumor-driven metabolic remodeling. Many genetic alterations associated with human cancers induce hyperproliferation of fly intestinal stem cells (ISCs) and lead to tumor formation in the midgut [17]. In particular, expression of constitutively active Yorkie (yki^3SA)—the Drosophila homolog of human YAP1—drives gut tumor development and triggers cachexia-like phenotypes, including lipid depletion, hyperglycemia, and wasting of muscle and ovaries [18, 19]. Mechanistically, yki^{3SA} gut tumors secrete multiple systemic factors, such as the IGF-antagonizing peptide *ImpL2* [18], *PDGF- and VEGF-related factor 1 (Pvf1*) [19] and unpaired 3 (*Upd3)* , a functional ortholog of mammalian interleukin-6 (IL-6) [20]. Notably, IL-6- and PDGF/VEGF-associated inflammatory programs are also broadly elevated across diverse cancer models [5-8]. Together, these observations highlight tumor–host organ communication as an evolutionarily conserved process and establish *Drosophila* as an excellent system to dissect tumor–host interactions *in vivo*."

7. It has been previously reported that muscle secretion of Pvf1 inhibits lipid synthesis in *Drosophila* oenocytes and regulates fat accumulation in the fat body by activating the Pi3K/Akt/TOR signaling pathway. This study focuses on how Pvf1 secreted by gut tumors promotes oenocytes lipid synthesis and thereby fuels tumor growth, which is contrary to the mechanism proposed in previous studies. Although the background is different (muscle homeostasis vs. tumor context), the authors do not clearly explain why Pvf1 plays such different roles in these two contexts. Also, since Pvf1 has been shown to regulate lipid accumulation in the fat body, it is important that the authors rule out the possibility that the lipid changes observed in Yki flies originate from alterations in the fat body rather than oenocytes.

-We think there might be tissue specific effects from muscle Pvf1 and gut Pvf1, as demonstrated in previous publication [21]. It was shown that only Pvf1 from the muscle inhibits lipid synthesis in the oenocyte and fat body, whereas Pvf1 from the fat body and midgut had no effects on oenocyte steatosis [21]. How is gut tumor derived Pvf1controlling oenocyte lipid metabolism has not been previously characterized. The mechanism of this tissue specificity might be from tissue specific post-translational modification of Pvf1, as PDGF/VEGF ligands are disulfide-linked dimers [22, 23]. The expression of Pvf2 and 3 is highly induced in gut tumors [20]. Although not yet demonstrated for *Drosophila* PVFs, PDGF/VEGF-family ligands in other systems can form distinct homo- and heterodimers that alter receptor signaling outputs [24]; thus, PVF heterodimerization remains a plausible mechanism that could diversify downstream responses .
-mElo expression is highly enriched in oenocytes and is strongly upregulated in oenocytes from Yki flies, whereas only minimal changes are observed in the fat body. This is supported by our single-nucleus RNA-seq analysis across multiple tissues from control and Yki flies, in which CG18609 corresponds to mElo.

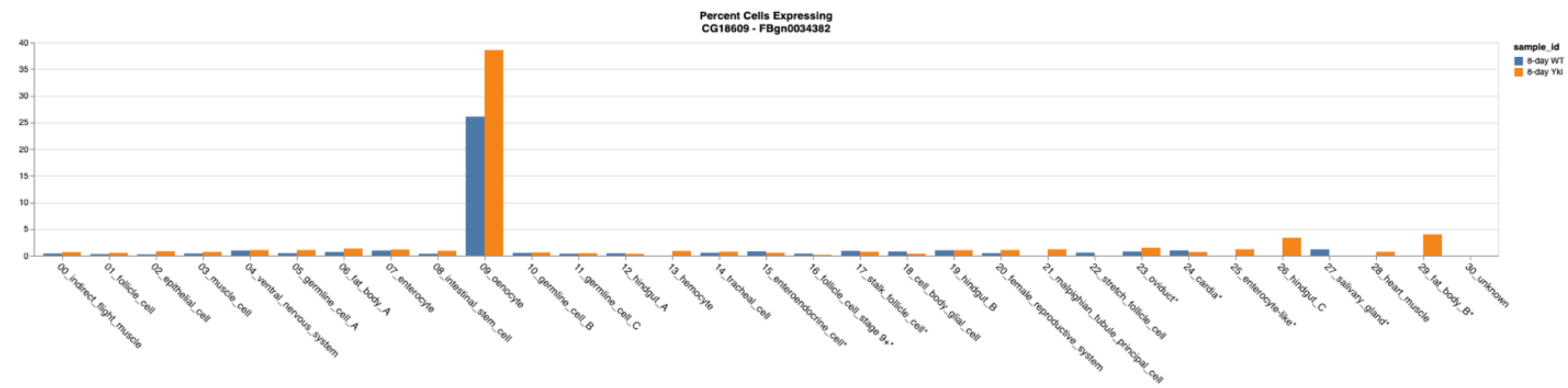

Knockdown of Hnf4 and mElo in the fat body did not affect gut tracheal morphology (Figure S6H–M). In contrast, simultaneous reduction of these genes in oenocytes led to a significant decrease in gut tracheal growth (Figure 6E–P). This data was added to the main manuscript (line 378-384).
These data suggest that oenocytes play a more prominent role than the fat body in regulating tracheal growth.

Minor concerns:
1. Scale bars in figures lack units in the legends. The authors should indicate the units for each figure to ensure clarity and reproducibility.
We thank reviewer for this suggestion, and we modified the manuscript accordingly.

2. The lipidomic profiling data in figures use "Normalized area" as the unit (e.g., Figure 1J). This term is ambiguous and should be clarified (e.g., normalized to total protein, internal standards, or sample volume) to ensure accurate interpretation.
We changed "normalized area" to "normalized intensity". Normalized intensity was calculated as the integrated LC–MS peak area for each lipid species, normalized to the sample's total signal (total reads) and to the analyzed sample volume.

3. The Y-axis label in Figure 1J appears to omit "x106" or a similar scaling factor, making the data scale unclear. The authors should verify and correct the axis label.
We have modified accordingly.

4. The legends for Figure 1K and 1L are reversed.
New figures are arranged.

5. The method for measuring ovary size is inconsistent between Figure S1K (unit: Relative area) and S1L (unit: ovary size). The authors should standardize the measurement method and units across panels and provide a detailed description in the methods.
We have changed all the ovary measurement to "ovary size" instead of "relative area".

6. The figure legend for Figure 4 omits the label of "Fig. 4A".
We adjusted this accordingly.

7. In Figure 4I-K, the authors compare Esg>Luc-i (control) versus Esg>yki,LpR1-i or Esg>yki,LpR2-i. Why are Esg>yki,Luc-i flies not used as a control to assess the specific effects of LpR1/LpR2 knockdown in the context of intestinal tumors?

We appreciate reviewer's comment, and we have modified the comparison accordingly.

1. Adamson, A.W., et al., *Hepatocyte nuclear factor-4alpha contributes to carbohydrate-induced transcriptional activation of hepatic fatty acid synthase.* Biochem J, 2006. **399**(2): p. 285-95.
2. Bideyan, L., R. Nagari, and P. Tontonoz, *Hepatic transcriptional responses to fasting and feeding.* Genes Dev, 2021. **35**(9-10): p. 635-657.
3. Hirota, K., et al., *A Combination of HNF-4 and Foxo1 Is Required for Reciprocal Transcriptional Regulation of Glucokinase and Glucose-6-phosphatase Genes in Response to Fasting and Feeding* *. Journal of Biological Chemistry, 2008. **283**(47): p. 32432-32441.
4. Palanker, L., et al., *Drosophila HNF4 regulates lipid mobilization and beta-oxidation.* Cell Metab, 2009. **9**(3): p. 228-39.
5. Konishi, T., et al., *The K-ras gene regulates vascular endothelial growth factor gene expression in non-small cell lung cancers.* Int J Oncol, 2000. **16**(3): p. 501-11.
6. Sunaga, N., et al., *Targeting Oncogenic KRAS in Non-Small-Cell Lung Cancer.* Cancers (Basel), 2021. **13**(23).
7. Brooks, G.D., et al., *IL6 Trans-signaling Promotes KRAS-Driven Lung Carcinogenesis.* Cancer Res, 2016. **76**(4): p. 866-76.
8. Olivares-Hernández, A., et al., *Current and future perspectives in clinical practice in KRAS-mutated non-small cell lung cancer: a literature review.* Precision Cancer Medicine, 2024. **7**.
9. Liu, Y., et al., *Hepatic gluconeogenesis and PDK3 upregulation drive cancer cachexia in flies and mice.* Nat Metab, 2025. **7**(4): p. 823-841.
10. Billeter, J.C., et al., *Specialized cells tag sexual and species identity in Drosophila melanogaster.* Nature, 2009. **461**(7266): p. 987-91.
11. Huang, K., et al., *Impaired peroxisomal import in Drosophila oenocytes causes cardiac dysfunction by inducing upd3 as a peroxikine.* Nat Commun, 2020. **11**(1): p. 2943.
12. Xu, J., et al., *Mechanistic characterization of a Drosophila model of paraneoplastic nephrotic syndrome.* Nat Commun, 2024. **15**(1): p. 1241.
13. Tamamouna, V., et al., *Remodelling of oxygen-transporting tracheoles drives intestinal regeneration and tumorigenesis in Drosophila.* Nat Cell Biol, 2021. **23**(5): p. 497-510.
14. Sargent, J.R., R.R. Gatten, and R. McIntosh, *Wax esters in the marine environment — their occurrence, formation, transformation and ultimate fates.* Marine Chemistry, 1977. **5**(4): p. 573-584.

15. Rosca, M.G., et al., *Oxidation of Fatty Acids Is the Source of Increased Mitochondrial Reactive Oxygen Species Production in Kidney Cortical Tubules in Early Diabetes.* Diabetes, 2012. **61**(8): p. 2074-2083.
16. Simcox, J., et al., *Global Analysis of Plasma Lipids Identifies Liver-Derived Acylcarnitines as a Fuel Source for Brown Fat Thermogenesis.* Cell Metabolism, 2017. **26**(3): p. 509-522.e6.
17. Patel, P.H. and B.A. Edgar, *Tissue design: How Drosophila tumors remodel their neighborhood.* Seminars in Cell & Developmental Biology, 2014. **28**: p. 86-95.
18. Kwon, Y., et al., *Systemic organ wasting induced by localized expression of the secreted insulin/IGF antagonist ImpL2.* Dev Cell, 2015. **33**(1): p. 36-46.
19. Song, W., et al., *Tumor-Derived Ligands Trigger Tumor Growth and Host Wasting via Differential MEK Activation.* Dev Cell, 2019. **48**(2): p. 277-286 e6.
20. Song, W., et al., *Tumor-Derived Ligands Trigger Tumor Growth and Host Wasting via Differential MEK Activation.* Developmental Cell, 2019. **48**(2): p. 277-286.e6.
21. Ghosh, A.C., et al., *Drosophila PDGF/VEGF signaling from muscles to hepatocyte-like cells protects against obesity.* eLife, 2020. **9**: p. e56969.
22. Hoch, R.V. and P. Soriano, *Roles of PDGF in animal development.* Development, 2003. **130**(20): p. 4769-84.
23. Holmes, D.I.R. and I. Zachary, *The vascular endothelial growth factor (VEGF) family: angiogenic factors in health and disease.* Genome Biology, 2005. **6**(2): p. 209.
24. Heldin, C.H. and B. Westermark, *Mechanism of action and in vivo role of platelet-derived growth factor.* Physiol Rev, 1999. **79**(4): p. 1283-316.

Reviewer's Comments

Reviewer #1 (Remarks to the Author)

Overall, the authors have made a serious effort to address the reviewers' comments by adding new quantifications and supplementary data. However, the reorganization of the paper, particularly the repositioning of certain figures and arguments, is still somewhat difficult to follow. While several technical concerns have been adequately handled, important conceptual issues remain.

1. Although oenocytes may show relatively high Hnf4 positivity, the snRNA-seq indicates substantial Hnf4-positive fractions across multiple tissues, and the tumor-associated changes are not uniquely or consistently restricted to oenocytes. Moreover, the proportion of Hnf4-positive cells does not consistently change under Yki tumor conditions. Given that the manuscript discusses systemic phenotypes such as bloating, ovary size changes, and lifespan modulation, it is important to reconsider whether these organismal outcomes can be primarily attributed to the oenocyte–Hnf4–mElo axis. The current data do not exclude the possibility that changes in other Hnf4-expressing tissues contribute to these systemic phenotypes, and this limitation should be more explicitly acknowledged.

Thank you for this insightful comment. We agree that Hnf4 is also induced in other tissues, including the Malpighian tubules, fat body, and muscle. While our study does not exclude the possibility that tumor-associated Yki activity may elevate Hnf4 in these tissues and thereby contribute to systemic phenotypes (e.g., bloating, ovarian size changes, and reduced lifespan), the primary focus of this manuscript is the oenocyte Hnf4–mElo axis and its role in promoting intestinal tumorigenesis. In this context, the tumor-induced wasting-related phenotypes are considered secondary outcomes. We have included this limitation in the discussion section (518-521). Ongoing work in our lab, together with related efforts from others, is actively investigating how heightened Hnf4 in muscle and/or fat body may influence systemic wasting in the Yki tumor model.

2. The mechanistic basis for the selective and pronounced induction of mElo downstream of Hnf4 remains insufficiently resolved. The response largely provides speculative explanations rather than direct experimental evidence addressing target selectivity. As long as the main claim of this paper relies on this particular axis (related to point 1), obtaining more direct evidence supporting target selectivity (for example, ChIP-based or other regulatory evidence) would strengthen this part.

We thank the reviewer for this comment. Oenocyte-specific ChIP-based experiments are technically challenging because oenocytes are difficult to dissect in the large quantities required for ChIP analysis. However, we performed RT–PCR in oenocytes from Yki flies and found that reducing *mElo* expression (Fig. 1I) diminishes Hnf4-dependent transcriptional

output, supporting mElo as a transcriptional target of Hnf4 (at least indirectly). We also showed that Hnf4 nucleus localization increased under Yki condition, suggesting its increased activities (Figure 1E).

Although direct evidence of Hnf4 target selectivity would be informative, pursuing these experiments is beyond the scope of the current study and would detract from the central focus of the manuscript, which is to define how Hnf4-regulated lipid metabolism in oenocytes supports tumor growth.

Another possible explanation for this apparent target selectivity is the technical limitation of oenocyte dissection. Because oenocytes are closely associated with the fat body, some degree of tissue mixing during dissection is unavoidable. Since mElo is highly enriched in oenocytes, contamination from fat body tissue would be expected to have little to no effect on its measured expression. In contrast, other Hnf4 target genes, including Yip2, Hex-C, Acc, and Acady, are expressed at relatively high levels in both oenocytes and fat body, making their expression measurements more susceptible to fat body contamination. Therefore, the absence of a detectable change in expression does not necessarily indicate a lack of Hnf4 binding to these genes.

3. The proposed relay between oenocyte-derived lipids and tracheal remodeling remains the least well-supported part of the model. In particular, knockdown experiments with markedly different effect sizes (e.g., relatively weak versus strong phenotypes) are discussed in parallel and described in similarly strong terms. LpR1 knockdown appears weaker than LpR2 knockdown, with substantial overlap of data points. This may be somewhat overinterpreted and may lead to an overemphasis of the lipid uptake component. Clear differentiation of effect strength and a more proportionate interpretation are warranted.

We thank the reviewer for this comment. We agree that tracheal knockdown of *LpR1* did not reduce tumor proliferation (Fig. 4L). This may reflect differences in RNAi efficacy, as the *LpR2* RNAi line shows stronger knockdown than the *LpR1* RNAi line (Fig. S4F–G). In addition, *LpR2* expression is modestly higher in tracheal cells of Yki flies at day 8, which could explain why *LpR2* depletion produces a more pronounced phenotype. We have modified the main text accordingly (Line 366-369).

In summary, although the revision improves certain aspects of the manuscript, key conceptual and mechanistic gaps remain, and the framing of the systemic tumor–oenocyte–trachea circuit would benefit from a more cautious and consistent interpretation. These issues may be addressable through clearer framing, more cautious discussion, and appropriate limitation of claims, without necessarily requiring extensive new experiments.

Reviewer #2 (Remarks to the Author):

The revisions to this mauscript are mostly satisfactory. As before, the paper establishes an interesting set of cross-organ interactions between gut tumors, oenocytes, and trachea, that drives tumor growth. The model is coherent an appealing and supported by abundant genetic data, as well as some good lipidomics data, athough direct demonstrations of ligand (Pvf1) and lipid transport between the organs is not provided. The paper has been conscientiously edited to fix mistakes, and a modest amount of new data are included. As described below, there are still some mistakes in the text that need fixing, and a few overstated conclusions that should be qualified. The limitations of the study are not clearly articulated, and it would be helpful for the authors to do this, since alternative explanations of many of the genetic results are possible. Nevertheless, the authors present extensive and compelling evidence for their tumor-coenocyte-trachea-tumor interaction model, and it should be of interest to many readers. The remaining issues are minor, as listed below:

1. The sentence "...Our results also demonstrate that lipids produced..." (lines 31-32, Abstract) is an overstated conclusion that is not definitively warranted by the data. This statement should be changed to more accurately reflect the data, which show changes in hemolymph lipid constituents and alterations effected by depleting the lipoprotein receptor LpR2 in each of the three organs studies. Lipid transport is not directly assessed.

We thank the reviewer for this comment. We modified the abstract accordingly: “Lipoprotein-mediated lipid transport and altered oenocyte lipid metabolism drive the observed changes in tracheogenesis.”

2. Regarding lipid transport, it is surprising that the lipoprotein lipid transporter, Lpp, is not subjected to testing here. Would it not be quite interesting and informative to suppress cross-organ lipid transport by Lpp knockdown? Please comment.

We appreciate the reviewer's insightful comment. Apolipophorin (apolpp/Lpp), the major hemolymph lipoprotein in flies, transports neutral lipids, phospholipids, and sterols between tissues and is produced exclusively in the fat body [1, 2]. However, due to its fat body enrichment and our focus on oenocyte–trachea–gut tumor interactions, we prioritized oenocyte- or trachea-specific knockdown of *LpR1* and *LpR2* to highlight the roles of these tissues in tumor growth.

3. Some of the authors' discussion points as made in the rebuttal seem not to have been added to the paper proper. Please review the manuscripts to ensure that important points of discussion from the rebuttal are incorporated into the revised text. For instance, the points made in response to Reviewer #2 questions 7 and 9, and Reviewer #3 question 7, would be helpful also to mention in the paper's main text.

We thank the reviewer for this thoughtful comment. Since Reviewer #2 questions 7 and 9 address the similar problem, we summarized the answers from the two and included in the discussion section (Line 500 – 520):

“Our data indicate that wax esters (WEs) and acylcarnitines (AcCas) are elevated in tumor-bearing flies and mice (Figure 1A and 7N). Notably, in both models we observe an increase in WEs containing very-long-chain fatty acids (VLCFAs), consistent with the elevated expression of Hnf4 and fatty acid elongases in the KL mouse liver as well as in oenocytes from Yki flies. Functionally, increased AcCas and WEs could contribute to tracheogenesis by supporting local energetic demand. For example, hepatocyte-derived plasma acylcarnitines have been shown to be required for systemic thermogenesis under cold exposure in mice, where peripheral tissues such as brown adipose tissue, skeletal muscle, and heart take up circulating acylcarnitines and oxidize them to fuel metabolism [55]. In addition, wax esters can serve as high-density neutral lipid reserves that are mobilized to meet energetic demands in diverse physiological contexts (e.g., diapause and reproduction in marine organisms). Alternatively, lipid elevation could promote tracheogenesis by increasing reactive oxygen species (ROS), which has been implicated as a driver of tracheal growth [42]. WEs can be hydrolyzed into fatty acids and fatty alcohols [42, 85]. VLCFAs released from WEs may undergo peroxisomal oxidation, generating H_2O_2 as a byproduct, which can stimulate tracheogenesis [42]. In parallel, fatty acids can be transported to mitochondria for β-oxidation, and high fatty-acid flux through mitochondrial respiration can increase electron leak from the ETC and elevate ROS production, potentially contributing to tracheogenesis [86]. These studies also leave open the possibility that metabolites derived from these lipid species act as signaling molecules that promote tracheogenesis.”

We have added the following to the discussion section to address comments from reviewer #3 question 7 (Line 480-491):
“Previous studies showed that muscle-derived Pvf1 suppresses lipid synthesis in *Drosophila* oenocytes and limits fat accumulation in the fat body through PI3K/Akt/TOR signaling [50]. In contrast, our results indicate that Yki tumor–derived Pvf1 promotes very-long-chain fatty acid synthesis in oenocytes, thereby supporting tumor growth. This difference may reflect tissue-specific effects of Pvf1. Notably, muscle-derived Pvf1 was reported to inhibit oenocyte lipid synthesis, whereas Pvf1 from the fat body and midgut had no detectable effect on oenocyte lipid metabolism or steatosis [50]. How does tumor–derived Pvf1 regulate oenocyte lipid metabolism has not been previously examined. One possible explanation is that tissue-specific post-translational modification of Pvf1 alters its activity, as PDGF/VEGF ligands function as disulfide-linked dimers [75, 76]. In addition, Pvf2 and Pvf3 are strongly induced in gut tumors [22], raising the possibility that distinct PVF homo- or heterodimers may generate different downstream signaling outputs [53].”

4. There is some sloppy text in the Results section. For instance what are "Yki oenocytes" (lines 187, 188)? Which cells are referred to on line 279, for Hnf4 levels?
We thank the reviewers for the detailed suggetions.
We have modified the text accordingly.
“Yki oenocytes” are changed to “oenocytes of Yki flies”.
“Hnf4 levels” are changed to “Hnf4 levels in oenocytes”.

5. The results described on lines 333-335, demonstrating a lack effect of Hnf4 depletion in oenocytes, are presented (Fig S4A), but not explained. As these results are inconsistent with the authors' model (Fig 7o) they should be discussed.

We thank the reviewer for this comment. We have modified the text accordingly:
"The transport of lipids, such as phospholipids, from oenocytes to the trachea or ISCs to promote tumor growth has not been reported [3]. To test whether changes in oenocyte metabolism regulates ISCs growth, we inhibited Hnf4 activity in oenocytes by overexpressing *TSC1,2*, knocking down or overexpressing *Hnf4*, and observed no significant effect on the number of pH3+ cells in the midgut (Figure S4A). This suggests that changes in oenocyte lipid metabolism does not promote ISC proliferation under normal condition."
These observations are consistent with our model in which alterations in lipid profiles caused by oenocyte-specific manipulation of *Hnf4* or knockdown of lipophorin receptors regulate tracheal growth, rather than directly affecting ISC proliferation, under both basal (Figure S4A) and tumor conditions (Figure S4F). Thus, ISC expansion does not appear to depend directly on lipid metabolism, but instead on tracheogenesis driven by signals from peripheral tissues.

6. Some of the Figure citations are wrong (e.g. lines 204, 280, 281, 284).
We applogize for the mistake. We have fixed the errors and proofread to eliminate additional mistakes.

Reviewer #3 (Remarks to the Author):

The authors have attempted to address most of the concerns that were highlighted in the original version of the manuscript, with some additional experimentation and substantial changes to the text. In general, the overall findings are intriguing, amalgamating tumor signaling mechanisms, distinct aspects of lipid metabolism, and tracheogenesis/angiogenesis to generally support a model of tumor-host interactions in Drosophila. Mechanistically, data support a signaling module by which tumor-secreted Pvf1 (PDGF/VEGF-like factor) activates the TORC1-Hnf4 pathway in lipid-producing oenocytes, leading to increased production of unique lipid species that are likely needed for tracheogenesis.

The revisions have, to an extent, improved the manuscript. However, I have a few comments which do not preclude publication of the manuscript.

First, there are numerous (too many to list) mistakes in the text/Results Section in which the description of data or a reference to a specific Figure panel does not match the actual data/Figure. This includes references in the text to Fig. S1H-I, Fig. 1H, Fig. S1J, Fig. S1K, Fig. S2M and S2D, Fig. 3G-H, and many more. These mismatches make the manuscript extremely difficult to read and evaluate. In general, the organization and presentation of the data and concepts inhibit readability.

We applogize for the mistake. We have fixed the errors and proofread to eliminate additional mistakes.

Secondly, even with the revisions, it is still challenging to conceptualize the Pvf1-dependent signaling axis proposed in the manuscript, especially when considering previous findings. Pvf1 has such diverse effects on adult fly metabolism (both in tumor and non-tumor contexts), and new findings presented here suggest even context-specific opposing effects on lipid metabolism within the same tissues, but it's not clear how a single ligand could achieve these effects (even if the secretion source is different). I think these types of paradigms limit the impact, at least mildly, of the new signaling axis described in the manuscript.
We thank the reviewer for this insight. Pvf1 does show tissue specific effects based on previous literature finding. Notably, muscle-derived Pvf1 was reported to inhibit oenocyte lipid synthesis, whereas Pvf1 from the fat body and midgut had no detectable effect on oenocyte lipid metabolism or steatosis [4]. In addition, we tried to overexpress *Pvf1* from both muscle tissues and intestinal stem cells in wild type flies, and observed opposite effects on oenocyte *mElo* expression (Figure 3L). This might indicate that the post-translational modification from different source tissues play a significant role in regulating Pvf1's function. More experimental data needed to address these differences and we are actively exploring these activities.

1. Prasad, S.V., et al., *Lipoprotein biosynthesis in the larvae of the tobacco hornworm, Manduca sexta.* J Biol Chem, 1986. **261**(36): p. 17174-6.
2. Palm, W., et al., *Lipoproteins in Drosophila melanogaster--assembly, function, and influence on tissue lipid composition.* PLoS Genet, 2012. **8**(7): p. e1002828.
3. Tamamouna, V., et al., *Remodelling of oxygen-transporting tracheoles drives intestinal regeneration and tumorigenesis in Drosophila.* Nat Cell Biol, 2021. **23**(5): p. 497-510.
4. Ghosh, A.C., et al., *Drosophila PDGF/VEGF signaling from muscles to hepatocyte-like cells protects against obesity.* Elife, 2020. **9**.
