# Supplementary material for: Lipid metabolism of hepatocyte-like cells supports intestinal tumor growth in Drosophila
Source: Nat Commun. 2026 Jul 3;17:8296. doi: 10.1038/s41467-026-75074-w (PMC13470496; doi:10.1038/s41467-026-75074-w)
Supplement: Supplementary file 4 [file 41467_2026_75074_MOESM2_ESM.pdf]

Description of Additional Supplementary Files:

Supplementary Data 1: Lists all genotypes of flies conducted in each experiment.

Supplementary Data 2: Unprocessed intensity reads for the lipidomic data extracted from whole female flies with control (*Esg-LexA>w),* Yki (*Esg-LexA>LexAop-Yki*), Yki;TSC1,2 (*Esg-LexA>LexAop-Yki*; *PromE-Gal4>UAS-TSC1,2)*, Yki;Hnf4-i (*Esg-LexA>LexAop-Yki*; *PromE-Gal4>UAS-Hnf4-i)*.

Supplementary Data 3: Unprocessed intensity reads for the lipidomic data extracted from hemolymph of female flies with control (*Esg-LexA>w),* Yki (*Esg-LexA>LexAop-Yki*), and Yki;Hnf4-i (*Esg-LexA>LexAop-Yki*; *PromE-Gal4>UAS-Hnf4-i)*.

Supplementary Data 4: List of names for lipidomics.

Supplementary Data 5: List of primer sequences used for RT-PCR analysis.

Supplementary Data 6: List of genes involved in very long chain fatty acid pathway.

Supplementary Data 7: Unprocessed intensity reads for the lipidomic data extracted from male mice (fed) and tumor bearing mice with cachexia (CACS).
